# Integrated torrefaction-anaerobic digestion of bamboo waste for enhanced energy recovery: process optimization, product characterization, and techno-economic evaluation

**DOI:** 10.1038/s41598-026-52760-9

**Published:** 2026-05-21

**Authors:** Himanshu Kachroo, Tharaka Rama Krishna C. Doddapaneni, Priyanka Kaushal, Sabine Kutschke, Rohan Jain

**Affiliations:** 1 School of Interdisciplinary Research, Indian Institute of Technology Delhi, Hauz Khas, New Delhi, 110016 India; 2 Helmholtz Institute Freiberg for Resource Technology, Helmholtz-Zentrum Dresden-Rossendorf, Bautzner Landstraße 400, 01328 Dresden, Germany; 3 Renewable and Sustainable Energy Research Center, Technology Innovation Institute, Masdar City, Abu Dhabi, United Arab Emirates; 4 Clean Energy Laboratory, Centre for Rural Development and Technology, Indian Institute of Technology Delhi, Hauz-Khas, New Delhi, 110016 India

**Keywords:** Bamboo waste, Circular bioeconomy, Torrefaction, Anaerobic digestion, Bio-coal and bio-methane, Techno-economic assessment, Energy science and technology, Engineering, Environmental sciences

## Abstract

This study evaluates an integrated valorization pathway for bamboo waste that combines torrefaction and anaerobic digestion (AD) to recover energy from both solid and liquid process streams within a circular economy framework. Specifically, a dual-stream valorization approach is developed to maximize energy recovery from both bio-coal and torrefaction condensate. Bamboo’s high lignin content (24–27 wt%), low ash content (< 3 wt%), and favorable volatile composition enable enhanced thermochemical upgrading and subsequent biochemical energy recovery. Torrefaction at 290 °C for 60 min increased the higher heating value (HHV) of bio-coal from 17.6 $\:\pm\:$ 0.4 MJ/kg to 25.4 $\:\pm\:$ 1.5 MJ/kg, accompanied by ~ 66% reduction in volatile matter and significant lignin enrichment, resulting in improved fuel quality. The aqueous condensate, typically underutilized, containing biodegradable organic acids with low inhibitor concentrations achieved a biomethane potential of 493 $\:\pm\:$ 1.7 mL-CH_4_/g-VS during AD, indicating its suitability as a secondary energy source under the tested conditions. The integrated process delivers a net energy recovery of approximately 21 GJ/ton, which is higher than rice husk and rice straw evaluated under identical optimized operating conditions, highlighting feedstock-dependent performance differences under similar conditions. A techno-economic assessment of a 50,000 t/y integrated torrefaction-AD pilot plant facility in India indicated economic feasibility with an internal rate of return (IRR) of 15.8% and a payback period of 6.5 years. Notably, the economic analysis is directly based on experimentally derived mass and energy balances, enhancing its practical relevance. By enabling near-complete utilization of bamboo residues, the proposed pathway supports the development of decentralized biorefineries, sustainable resource use, circular economy principles, and UN Sustainable Development Goals (SDGs) 7, 12, and 13.

**Supplementary Information:**

The online version contains supplementary material available at 10.1038/s41598-026-52760-9.

## Introduction

Global energy demand is rising rapidly, driven by population growth, industrialization, and urbanization, while continued reliance on fossil fuels exacerbates greenhouse gas emissions. Coal and natural gas emit approximately 90–110 and 50–60 g CO_2_-eq/MJ, respectively, compared to 10–30 g CO_2_-eq/MJ for bioenergy pathways such as biogas and biodiesel^1^. Meeting the targets of the Paris Agreement requires the rapid deployment of low-carbon emission energy technologies^2^. The International Renewable Energy Agency estimates that bioenergy could supply up to 60% of global energy demand by 2050, avoiding up to 37 gigatons of CO_2_ emissions annually^3^. Currently, bioenergy accounts for nearly half of global renewable energy consumption, underscoring its pivotal role in the decarbonization transition.

Bamboo biomass offers unique advantages in this context due to its high lignocellulosic content, rapid growth, low ash (~ 3 wt%), and wide availability in tropical and subtropical regions^4^. India has ~ 14 million hectares under bamboo cultivation, producing ~ 14.6 million tonnes annually, with over 50% concentrated in the northeastern states^5^. However, processing inefficiencies in industries such as furniture, flooring, handicrafts, and incense stick manufacturing can reach up to 80%, generating 2.1–4.9 million tonnes of bamboo waste annually^6^. Despite this abundance, bamboo waste remains underutilized for energy recovery in India, unlike rice husk and rice straw, which dominate biomass utilization in the Indo-Gangetic plains despite their high ash (~ 17–18 wt%), elevated silica levels, and associated slagging issues^7,8^. Bamboo’s low ash, higher energy density, and regional availability position it as a strategic feedstock for decentralized bioenergy production, particularly in bamboo-rich areas of Northeast India.

Torrefaction is a mild thermochemical pretreatment conducted under inert conditions at 200–300 °C, which improves biomass fuel quality by reducing volatile matter, increasing fixed carbon, and enhancing hydrophobicity and grindability^9,10^. The resulting bio-coal can achieve energy densities comparable to lignite or sub-bituminous coal^11–13^, while requiring lower capital investment and operating temperatures than pyrolysis or gasification^12^. However, torrefaction also produces a condensable volatile fraction, referred to as the torrefaction condensate, which is often underutilized or discarded due to its high water content, acidity, and presence of corrosive compounds^13,14^. Notably, this condensate contains biodegradable organics (acetic, formic, propionic, and lactic acids) alongside low concentrations of inhibitory compounds. Despite this, most existing studies primarily focus on solid product enhancement, with comparatively limited attention given to valorization of liquid by-products generated during torrefaction.

AD offers a promising pathway to convert these organic compounds into biomethane, enabling energy recovery from the liquid fraction. Previous studies have investigated AD of bamboo biomass^15^, as well as torrefaction condensates from feedstocks such as pine and rice husk, reporting methane yields of 470–510 mL-CH_4_/g-VS (volatile solids)^13,16^. However, integrated approaches that combine torrefaction and AD for bamboo waste remain largely unexplored. In particular, studies addressing the simultaneous valorization of both solid and liquid streams within a unified framework are scarce, limiting the overall efficiency and circularity of biomass conversion systems.

In our previous work^16^, the techno-economic feasibility of an integrated torrefaction-AD system was demonstrated for rice husk and rice straw. However, bamboo exhibits distinct physicochemical characteristics, including lower ash content, higher lignin fractions, and region-specific availability, which can significantly influence thermal degradation behavior, product distribution, and downstream biodegradability. Despite these differences, direct experimental comparisons across biomass types are often lacking, as most studies rely on disparate operating conditions or literature-reported data. Establishing a consistent comparative framework is therefore essential for generating reliable feedstock-specific insights and informing process optimization and scale-up.

Despite recent advances, to the best of our knowledge, limited studies have demonstrated an integrated torrefaction-AD framework for bamboo waste that simultaneously valorize both solid and liquid streams while enabling controlled comparison with conventional residues under identical operating conditions. Furthermore, the integration of experimental results with mass-energy balance and techno-economic evaluation within a single framework remains limited, restricting the translation of laboratory-scale findings into deployable bioenergy systems.

To addresses these gaps, the present study provides the comprehensive evaluation of bamboo waste within an integrated torrefaction-AD platform, encompassing: (i) optimization and characterization of bamboo-derived bio-coal; (ii) AD of bamboo-derived torrefaction condensate for biomethane production; (iii) direct benchmarking against rice husk and rice straw under identical operating conditions; and (iv) a detailed techno-economic analysis for a 50,000 t/y integrated torrefaction-AD facility in the Indian context. By combining dual-stream valorization, controlled comparative analysis, and system-level economic evaluation, this work advances the development of decentralized, feedstock-specific biorefineries, and provides a scalable pathway for complete biomass utilization within a circular bioeconomy framework.

## Results

### Bamboo waste characterization

The physicochemical characteristics of untreated bamboo waste were comprehensively characterized in a previous study by Kachroo et al.^17^. The initial moisture content of the raw bamboo was ~ 8 wt%, which was reduced to ~ 2 wt% after oven drying before analysis. Proximate analysis showed 16.0 $\:\pm\:$ 0.4 wt% fixed carbon, 74.0 $\:\pm\:$ 2.7 wt% volatile matter, and 2.0 $\:\pm\:$ 0.2 wt% ash^17^. Elemental analysis revealed 45.0 $\:\pm\:$ 1.7 wt% atomic carbon and 47.0 $\:\pm\:$ 0.8 wt% oxygen content^17^. The HHV was 17.6 $\:\pm\:$ 0.4 MJ/kg, and chemical composition included 49.0 $\:\pm\:$ 1.1 wt% cellulose, 24.0 $\:\pm\:$ 0.6 wt% lignin, and 23.0 $\:\pm\:$ 1.6 wt% hemicellulose, underscoring its potential as a promising feedstock for torrefaction^17^.

### Analysis of torrefaction products

**Fig. 1:**
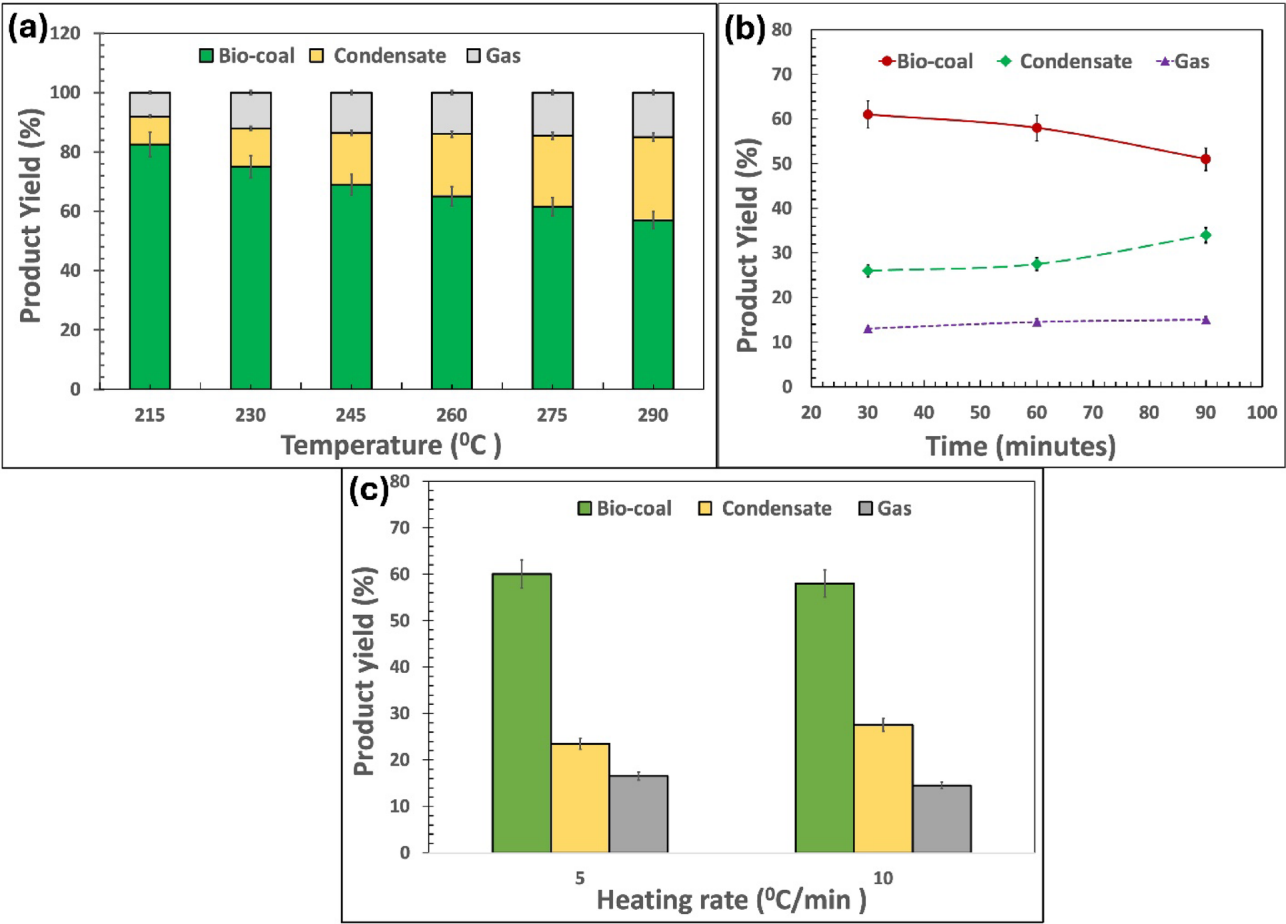
Torrefaction product yield distribution at (**a**) different torrefaction temperatures with residence time: 60 min, heating rate: 10 °C/min, (**b**) different residence times with temperature: 290 °C, heating rate: 10 °C/min and (**c**) different heating rate with temperature: 290 °C, residence time: 60 min.

Figure 1 presents the product yield distributions of bamboo waste under varying torrefaction temperatures and residence times, with error bars representing the standard deviation of the mean. The highest bio-coal yield was observed at 215 °C, after which it reduced from 83.0 $\:\pm\:$ 0.5 wt% to 58.0 $\:\pm\:$ 0.2 wt% as the torrefaction temperature increased from 215 °C to 290 °C (Fig. 1a). Extending the residence time from 30 to 90 min further reduced the bio-coal yield, ranging from 63.0 $\:\pm\:$ 0.7 wt% to 51.0 $\:\pm\:$ 0.5 wt% (Fig. 1b). Additionally, an increase in the heating rate from 5 °C/min to 10 °C/min resulted in an approximate 5 wt% variation in bio-coal yield.

The observed reduction in bio-coal yield with increasing temperature and residence time can be attributed to the progressive thermal degradation of biomass constituents. Hemicellulose, which is thermally least stable, decomposes within the torrefaction temperature range (200–260 °C), leading to significant mass loss through devolatilization and the formation of condensable volatiles and permanent gases (CO, CO_2_). As the temperature approaches 270–300 °C, partial depolymerization of cellulose occurs, further contributing to volatile release via decarboxylation and decarbonylation reactions. In contrast, lignin exhibits greater thermal stability and degrades over a broader temperature range, leading to a relative enrichment in the solid fraction and increased atomic carbon content. Prolonged residence time enhances these decomposition reactions and promotes secondary cracking of intermediate products, thereby reducing the solid yield. This trend is consistent with the observed decrease in bio-coal yield and corresponding increase in volatile fractions reported in Fig. 1.

Conversely, the yield of torrefaction condensate exhibited a positive correlation with both increasing temperature (245–290 °C) and prolonged residence time (60–90 min), rising from 9.5 $\:\pm\:$ 0.4 wt% to 28.0 $\:\pm\:$ 0.9 wt% (Fig. 1). Similarly, the fraction of uncondensed volatiles increased with higher torrefaction temperatures (245–290 °C) and extended residence times (60–90 min) (Fig. 1).

A torrefaction temperature of 290 °C, residence time of 60 min, and a heating rate of 10 °C/min were selected as the processing conditions for bio-coal production. These conditions were chosen based on their reported effectiveness in enhancing carbonization, thereby, reducing the H/C and O/C atomic ratios while increasing lignin content. The selected conditions were intended to produce bio-coal with fuel properties comparable to sub-bituminous coal (HHV: 24–28 MJ/kg, H/C: <1.0, O/C: 0.3–0.5, lignin: 50–60 wt%), as discussed in subsequent sections.

### Torrefaction product characterization at best torrefaction conditions

#### Bamboo-derived bio-coal characteristics

**Fig. 2:**
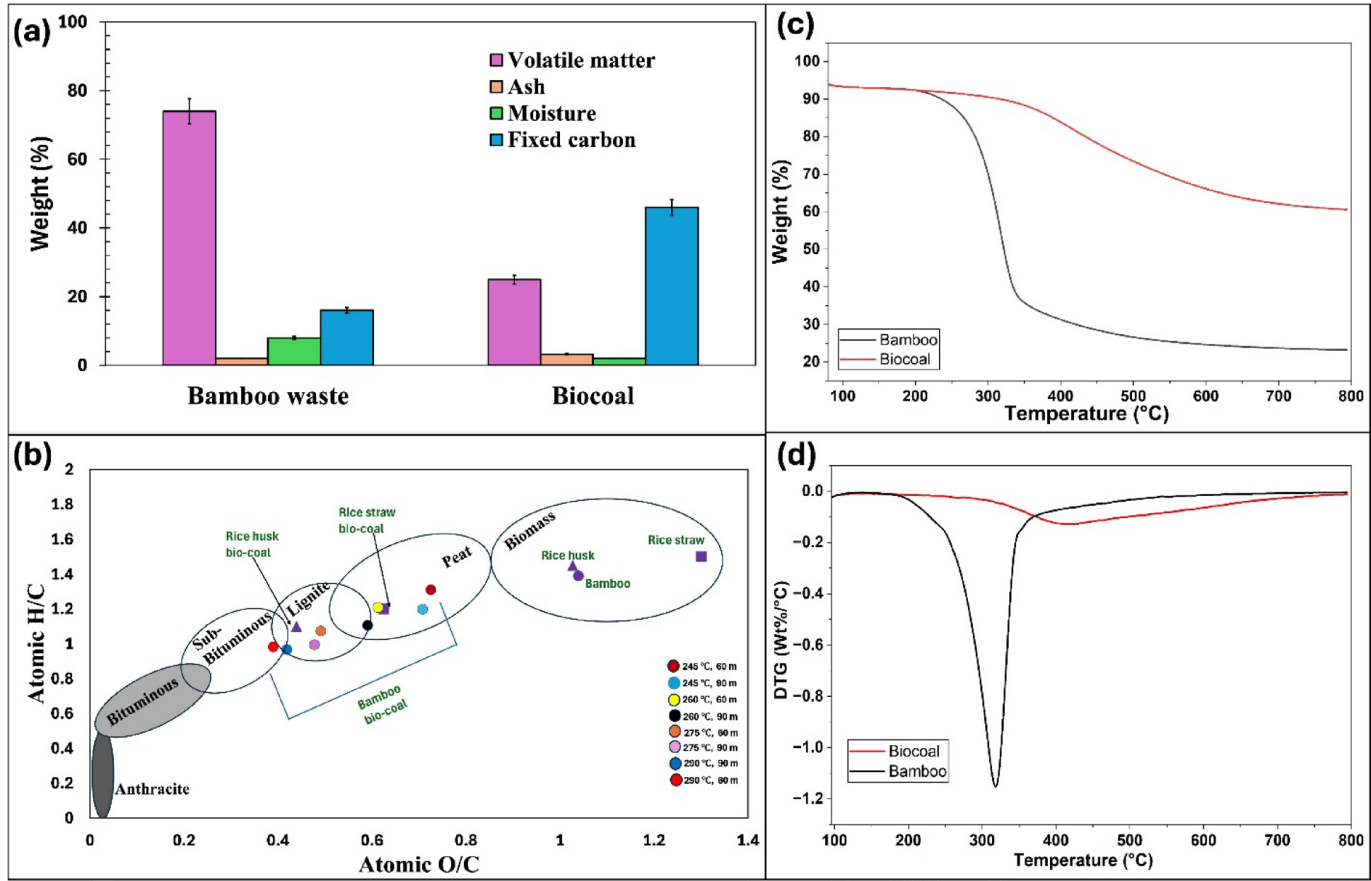
(**a**) Proximate analysis, (**b**) Van Krevelen diagram (H/C and O/C ratios are expressed on an atomic basis), (**c**) TGA curves, and (**d**) DTG curves of untreated and torrefied bamboo (at temperature: 290 °C, residence time: 60 min, and heating rate: 10 °C/min).

The proximate analysis indicated a reduction in volatile matter by 48.0 $\:\pm\:$ 0.4 wt% and moisture content by 6.0 $\:\pm\:$ 0.3 wt% (Fig. 2a). Conversely, the fixed carbon content increased by 29.8 $\:\pm\:$ 0.6 wt%, while the ash content exhibited a slight rise of 1.2 $\:\pm\:$ 0.4 wt% (Fig. 2a). Elemental analysis revealed a notable increase in atomic carbon content from 45.0 $\:\pm\:$ 0.7 wt% to 67.0 $\:\pm\:$ 0.6 wt%, alongside a decline in atomic oxygen content from 47.0 $\:\pm\:$ 0.3 wt% to 22.0 $\:\pm\:$ 0.8 wt%, respectively. The Van Krevelen diagram (Fig. 2b) demonstrated a pronounced downward shift in the atomic H/C and O/C values (H/C < 1.0, O/C < 0.4) at a temperature of 290 °C, residence time of 60 min, and heating rate of 10 °C/min. This shift reflects the progression of key thermochemical reactions occurring during torrefaction. The reduction in the H/C ratio is primarily attributed to dehydration reactions, which remove hydrogen and oxygen as water, and with the increased aromatization of the carbon structure. This results in diagonal downward movement in the Van Krevelen space. In contrast, the decrease in the O/C ratio is governed by decarboxylation and decarbonylation reactions, releasing CO_2_ and CO, respectively, and leading to a more decline in O/C ratio. These reactions collectively drive the elimination of oxygenated functional groups and enhance carbon enrichment in the solid matrix.

Consequently, the bio-coal exhibits improved fuel characteristics, with the HHV of bamboo-derived bio-coal reached 25.0 $\:\pm\:$ 1.5 MJ/kg, corresponding to an energy densification of ~ 8 MJ/kg compared to raw bamboo. This progressive carbonization increases aromaticity, reduces polarity, and enhances hydrophobicity and combustion performance. The crude fibre analysis further supports these transformations, showing a substantial increase in lignin content (54.0 $\:\pm\:$ 1.8%), near-complete degradation of hemicellulose (99.0 $\:\pm\:$ 0.4% reduction), and a significant reduction in cellulose content (45.0 $\:\pm\:$ 2.6%).

Observations indicated that torrefied bamboo produced at lower temperature (200–245 °C), shorter residence time (30 min), and slower heating rate (5 °C/min) exhibited higher atomic H/C and O/C ratios, along with higher volatile matter, and hemicellulose content. Conversely, it demonstrated lower fixed carbon, resulting in reduced energy densities. In contrast, extending the residence time to 90 min yielded negligible variations in fuel characteristics compared to bamboo bio-coal produced at 60 min. The corresponding quantitative data for H/C, O/C, volatile matter, and crude fibre content (hemicellulose, cellulose, and lignin) across all experimental conditions are provided in SI (Table S1).

The thermal behavior of bamboo-derived bio-coal at a temperature of 290 °C, residence time of 60 min, and heating rate of 10 °C/min was analyzed using thermogravimetric (TGA) curves, as depicted in Fig. 2c. The TGA results revealed that the weight loss in bamboo bio-coal was ~ 40 wt% lower than that of untreated bamboo in the temperature range of 220–650 °C (Fig. 2c). The derivative thermogravimetric (DTG) curves (Fig. 2d) indicated a primary thermal decomposition peak for bamboo bio-coal at 315 °C. However, the major decomposition peak in untreated bamboo was more pronounced, indicating greater volatilization of organic matter.

**Fig. 3:**
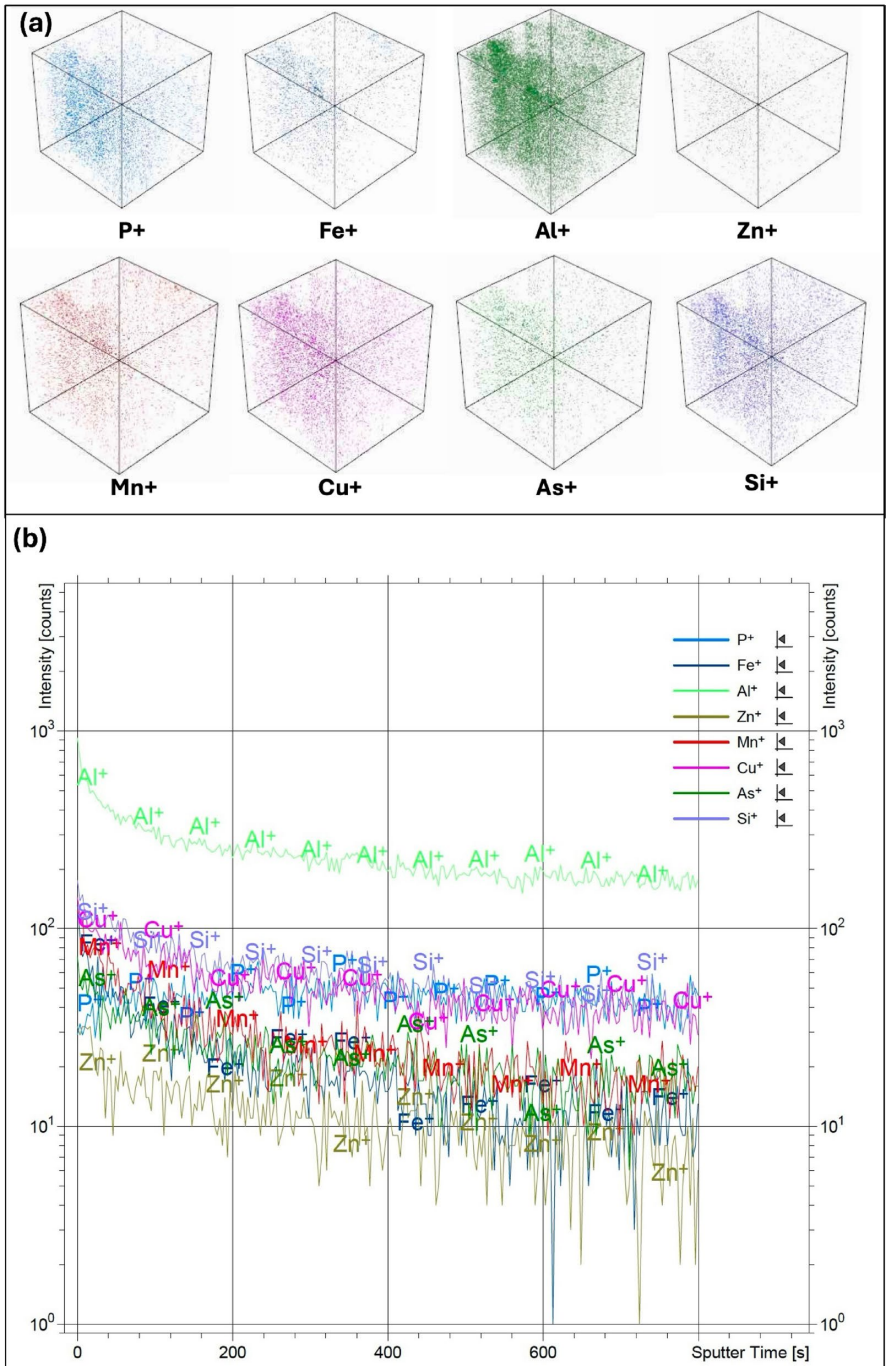
(**a**) Tof-SIMS 3D Render Overlay, and (**b**) Tof-SIMS depth profile of bamboo-derived bio-coal (at temperature: 290 °C, residence time: 60 min, and heating rate: 10 °C/min). Tof-SIMS ion images showing relative surface distribution of elements; data are qualitative and not to scale for concentration.

The distribution of inorganic trace metals near the surface and their depth-dependent variation in bamboo-derived bio-coal were investigated using time of flight-secondary ion mass spectrometry (Tof-SIMS). Depth profiling was conducted up to 500 μm to analyze the elemental distribution of Fe, Zn, P, Al, As, Si, Cu, and Mn in bio-coal produced under optimized torrefaction conditions, as shown in Fig. 3a. The depth profile (Fig. 3a) revealed a slight decrease in the concentration gradient and sputter ion intensities of trace metals from the surface to the deeper regions in the bulk of the bio-coal. Among the analyzed elements, Al exhibited the highest sputter ion intensity, followed by Si, Cu, Fe, Mn, As, P, and Zn (Fig. 3b).

The observed homogenous distribution of trace metals suggests enhanced mobility of inorganics during torrefaction, likely facilitated by the degradation of hemicellulose and cellulose fractions, which increases pore accessibility and promotes redistribution of inorganic constituents. This behavior is consistent with previous observations in rice husk-derived bio-coal^16,17^, indicating that thermal treatment can potentially plays a critical role in governing metal migration patterns.

From an application perspective, the relatively uniform dispersion of trace elements, combined with the low ash content (~ 3 wt%), is expected to improve combustion uniformity and reduce localized emissions. Furthermore, compared to rice husk-derived bio-coal, bamboo bio-coal exhibits lower concentrations of problematic inorganic species such as Si and Fe, thereby reducing the risk of slagging and fouling during high-temperature applications. This balance between elemental homogeneity and reduced inorganic load highlights the suitability of bamboo bio-coal as a cleaner and more efficient solid fuel for industrial applications.

It is important to note that Tof-SIMS is inherently a qualitative technique; therefore, the reported ion intensities represent relative elemental distribution rather than absolute concentrations due to matrix effects and variations in ionization efficiency. Furthermore, positive secondary elemental ions released from the bio-coal during sputtering were mapped as 3D SIMS images. The 3D elemental distribution map demonstrated a homogenous dispersion of trace metals across the sputter area (300 × 300 µm^2^), as depicted in Fig. 3b.

#### Bamboo-derived condensate characteristics

The torrefaction condensate derived from bamboo waste comprises a complex mixture of water-soluble and water-insoluble compounds, which were quantified by gas chromatography-mass spectrometry (GC-MS) and are presented in Table 1. Acetic acid was identified as the predominant organic constituent in the condensate, while water constituted the major fraction (Table 1). The condensate exhibited acidic characteristics, with a pH of 2.0 $\:\pm\:$ 0.2, a volatile solid content of 16.0 $\:\pm\:$ 0.7 wt%, and a HHV of 11.4 $\:\pm\:$ 0.3 MJ/kg.

**Table 1:** Composition of bamboo-derived condensate at temperature: 290 °C, residence time: 60 min, heating rate: 10 °C/min.

Chemical compounds	Composition (wt%)
*Organic acids*	
Acetic acid	3.79 $\:\pm\:$ 0.02
Lactic acid	1.42 $\:\pm\:$ 0.03
Formic acid	1.67 $\:\pm\:$ 0.05
Propanoic acid	0.81 $\:\pm\:$ 0.03
Butyric acid	0.69 $\:\pm\:$ 0.01
Benzoic acid	0.52 $\:\pm\:$ 0.03
*Aldehydes*	
3-Furaldehde	0.29 $\:\pm\:$ 0.06
Furfural	0.25 $\:\pm\:$ 0.07
5-hydroxymethylfurfural	0.09 $\:\pm\:$ 0.04
Methanol	1.24 $\:\pm\:$ 0.02
Phenol	0.12 $\:\pm\:$ 0.02
Water (%)	69.00 $\:\pm\:$ 2.51
Others	20.11 $\:\pm\:$ 1.68

#### Bamboo-derived uncondensed volatiles characteristics

**Fig. 4:**
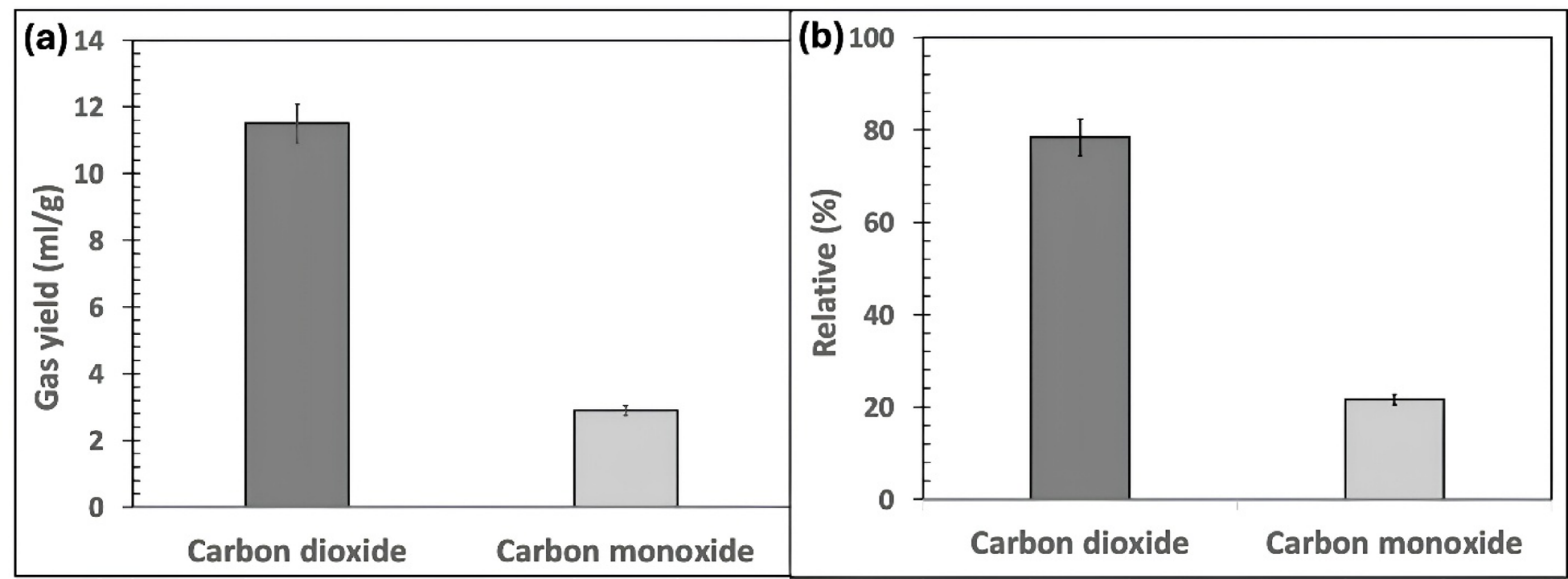
The (**a**) yield and (**b**) composition of uncondensed gases from bamboo waste torrefaction at temperature: 290 °C, residence time: 60 min, and heating rate: 10 °C/min.

The composition and yield of uncondensed volatiles generated during bamboo torrefaction conditions of temperature of 290 °C, residence time of 60 min, and heating rate of 10 °C/min were analyzed and quantified using GC-TCD (thermal conductivity detector), as illustrated in Fig. 4. The production of uncondensed volatiles was relatively low, ranging from 10 to 12 mL/g-biomass (Fig. 4a). CO_2_ and CO were identified as the predominant gaseous by-products, with minor traces (< 1%) of H_2_ detected (Fig. 4b).

**Fig. 5:**
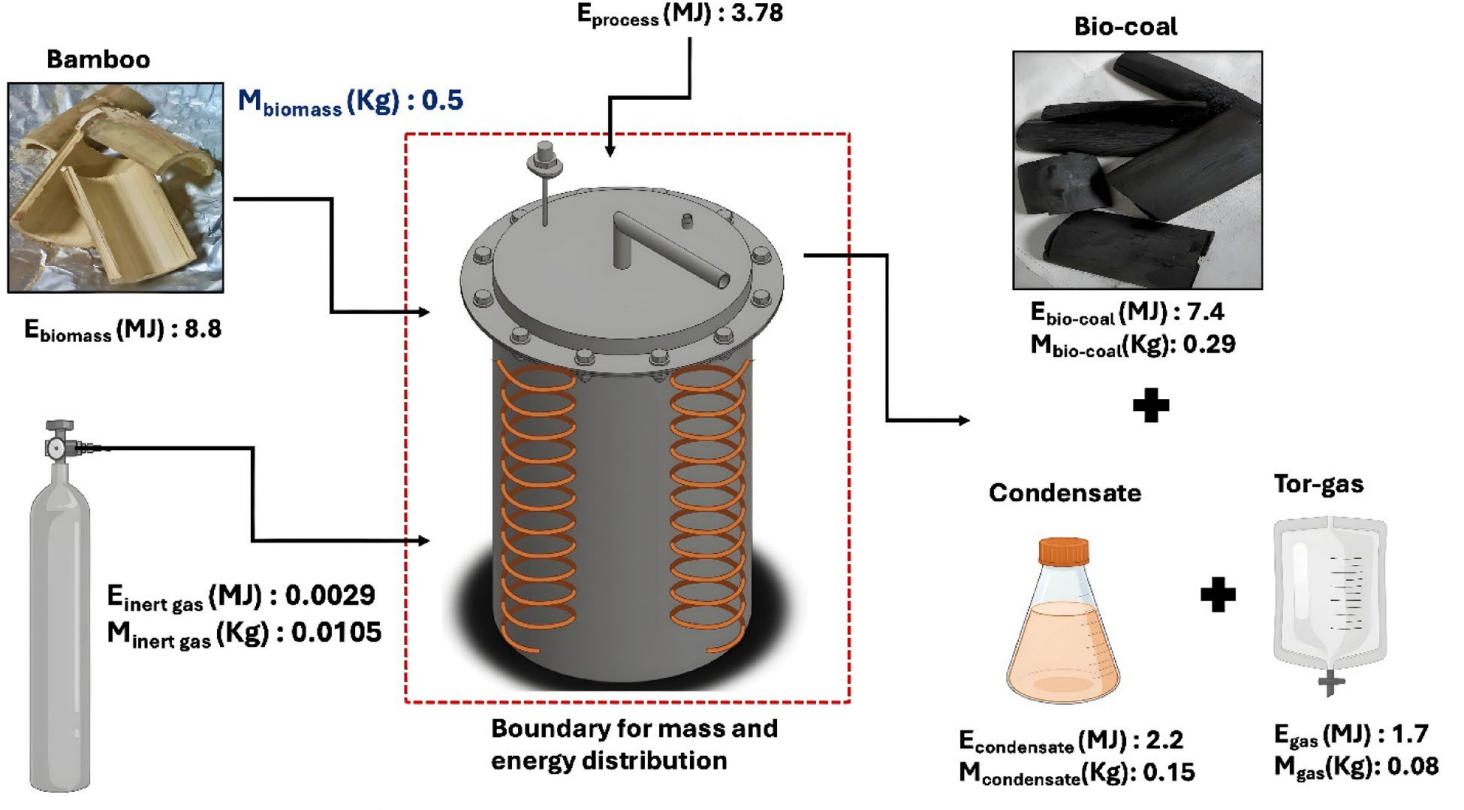
Mass and energy distribution of the bamboo torrefaction system at temperature: 290 °C, residence time: 60 min, and heating rate: 10 °C/min.

### Energy distribution of bamboo waste torrefaction

Energy distribution during bamboo waste torrefaction was evaluated at six different temperatures and three residence times (details in SI). The feedstock energy input remained constant across all runs, as each used a fixed mass of bamboo. With increasing torrefaction severity, the HHV of bamboo-derived bio-coal increased from 19.5 $\:\pm\:$ 1.1 MJ/kg to 25.5 $\:\pm\:$ 0.6 MJ/kg, reflecting carbon enrichment as volatiles were converted into condensates and gases. However, the overall energy yield of bamboo-derived bio-coal declined due to reduced mass yields at higher torrefaction severity. For example, at a 60-minute residence time, energy yield decreased from 93.0 $\:\pm\:$ 1.7% at 215 °C to 83.0 $\:\pm\:$ 0.8% at 290 °C. This decline was primarily attributed due to mass loss, not a reduction in energy density. Conversely, energy yields from torrefaction condensates and uncondensed gases increased with severity, indicating enhanced volatile release (SI) (Fig. 5). 

The mass and energy balance closures across all experimental conditions ranged from 95 to 99% and 98–99%, respectively, indicating good agreement between input and output streams (SI). Minor discrepancies in mass balance (1–5%) and energy balance (1–2%) can be attributed to experimental uncertainties, including condensate losses, limitations in gas measurement, and handling losses during sample collection.

### Bio-methane potential (BMP) of bamboo-derived torrefaction condensate

**Fig. 6:**
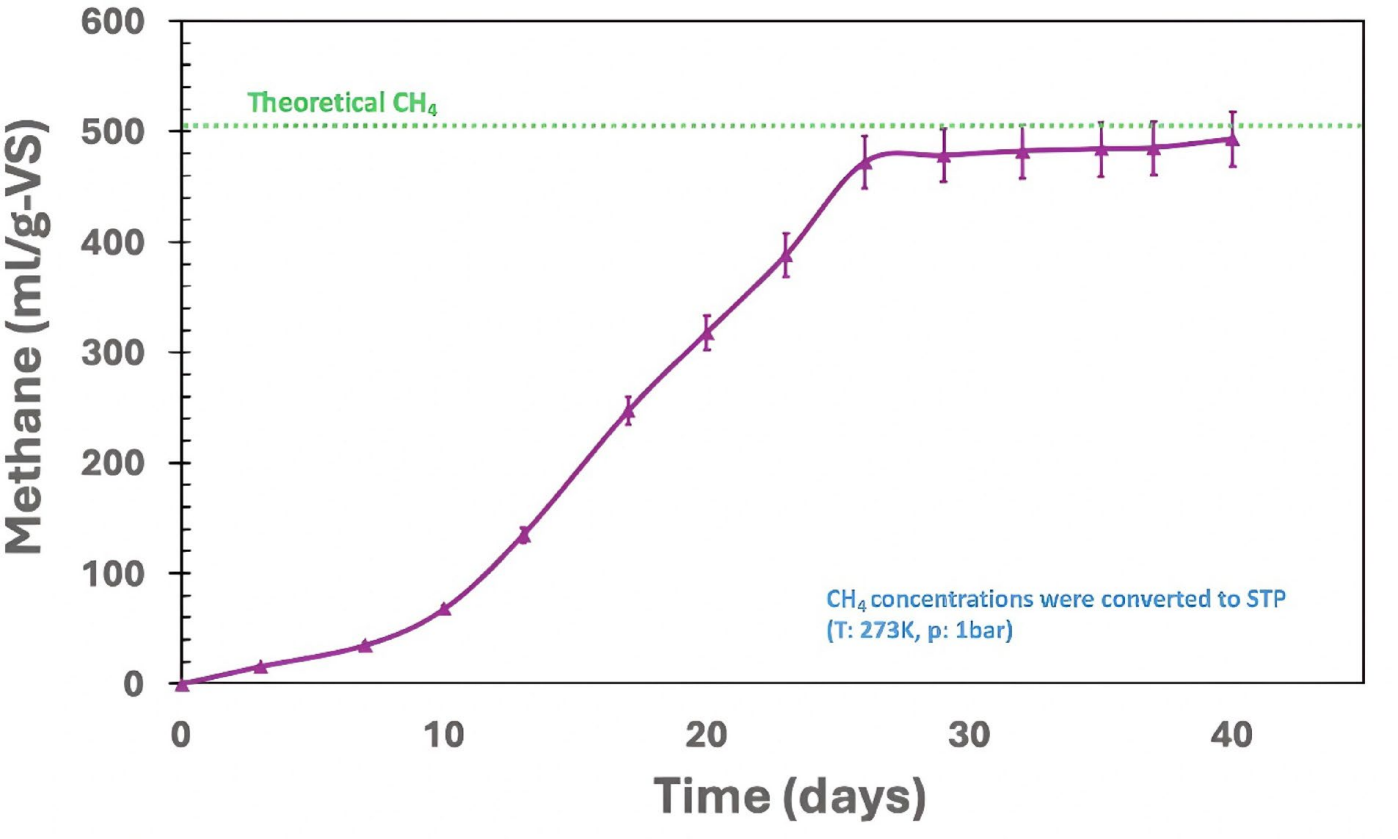
Cumulative methane yield during batch AD with bamboo waste-derived torrefaction condensate at 0.1 VS_substrate_: VS_inoculum_ loading and mesophilic conditions (37 $\:\pm\:$ 2 °C). Theoretical CH₄ (~ 500 mL/g-VS) was calculated using the Buswell equation based on the elemental composition of the condensate (C_1_H_2_O_0.83_N_0_).

The cumulative methane production during the AD of bamboo waste-derived torrefaction condensate under best selected conditions (temperature of 290 °C, heating rate of 10 °C/min, and residence time of 60 min) with a substrate-to-inoculum ratio of 0.1 (VS_substrate_: VS_inoculum_) at mesophilic temperatures for 40 days, is depicted in Fig. 6. The BMP assay yielded 493.0 $\:\pm\:$ 1.7 mL CH_4_/g-VS. A comparative analysis of volatile solids and chemical oxygen demand before and after AD is presented in Table 2. The total methane generated from the torrefaction condensate was 53.0 $\:\pm\:$ 0.3 mL (Table 2). Minimal variations in pH were observed following the BMP assay (Table 2). The VS content decreased by 31% after the AD, while the soluble chemical oxygen demand was reduced by 62% (Table 2).

**Table 2:** Volatile solids, and chemical oxygen demand before and after AD of bamboo waste-derived torrefaction condensate at temperature: 290 °C, residence time: 60 min, and heating rate: 10 °C/min.

Bamboo waste-derived torrefaction condensate (0.1VS_substrate_: VS_inoculum_)	Before inoculation	Final
pH	7.2 $\:\pm\:$0.2	7.1 $\:\pm\:$ 0.1
TS (%)	2.0 $\:\pm\:$ 0.1	1.8 $\:\pm\:$ 0.1
VS (%)	2.0 $\:\pm\:$ 0.1	1.3 $\:\pm\:$ 0.1
C: N:P ratio	30:1:1	–
Soluble chemical oxygen demand (g/L)	3.2 $\:\pm\:$ 0.4	2.0 $\:\pm\:$ 0.7
Total CH_4_ (mL)	–	53 $\:\pm\:$ 0.3

## Discussion

### Feedstock characteristics and process relevance

This study extends the integrated torrefaction-AD framework previously demonstrated for rice husk and rice straw^16^ to bamboo waste, a feedstock with improved lignocellulosic properties and distinct regional availability. However, beyond maintaining methodological consistency with prior work, the present study advances the framework by explicitly linking feedstock-specific physicochemical properties to coupled thermochemical-biochemical conversion performance.

Building on prior findings, this work highlights how intrinsic biomass characteristics govern process efficiency. Bamboo’s higher lignin content, lower ash fraction, and minimal silica-rich inorganic matrix significantly influence devolatilization behavior, carbon retention, and downstream biodegradability. These properties collectively position bamboo as a functionally advantageous feedstock within integrated bioenergy systems.

### Thermochemical transformations and bio-coal quality

Bamboo-derived bio-coal exhibits enhanced fuel characteristics, with HHV rising from 17.6 $\:\pm\:$ 0.4 MJ/kg to 25.5 $\:\pm\:$ 1.5 MJ/kg, positioning it as a viable replacement of sub-bituminous coal (Fig. 2b). This improvement is due to a ~ 66% reduction in volatile matter and an increase in fixed carbon content from 16.0 $\:\pm\:$ 0.4 wt% to 29.8 $\:\pm\:$ 0.6 wt% (Table S5).

From a mechanistic perspective, these enhancements arise from selective thermal degradation pathways: hemicellulose undergoes near-complete depolymerization, cellulose experiences partial chain scission, and lignin undergoes condensation and cross-linking reactions, forming a thermally stable aromatic matrix^10,11^. Notably, bamboo lignin exhibits relatively higher aromaticity and thermal stability compared to many agricultural residues (e.g., rice straw, bagasse)^10,11^. Its comparatively higher syringyl-to-guaiacyl (S/G) ratio is associated with lower cross-linking and moderate condensation, which can facilitate structural rearrangement and contribute to enhanced carbonization and energy densification during torrefaction. This behavior is consistent with reported lignin structural characteristics in bamboo, which influence its thermochemical conversion pathways.

These findings are consistent with previous reports on torrefied bamboo and woody biomass^10,18^, confirming the effectiveness of torrefaction in upgrading fuel quality.

### Condensate composition and AD performance

**Fig. 7:**
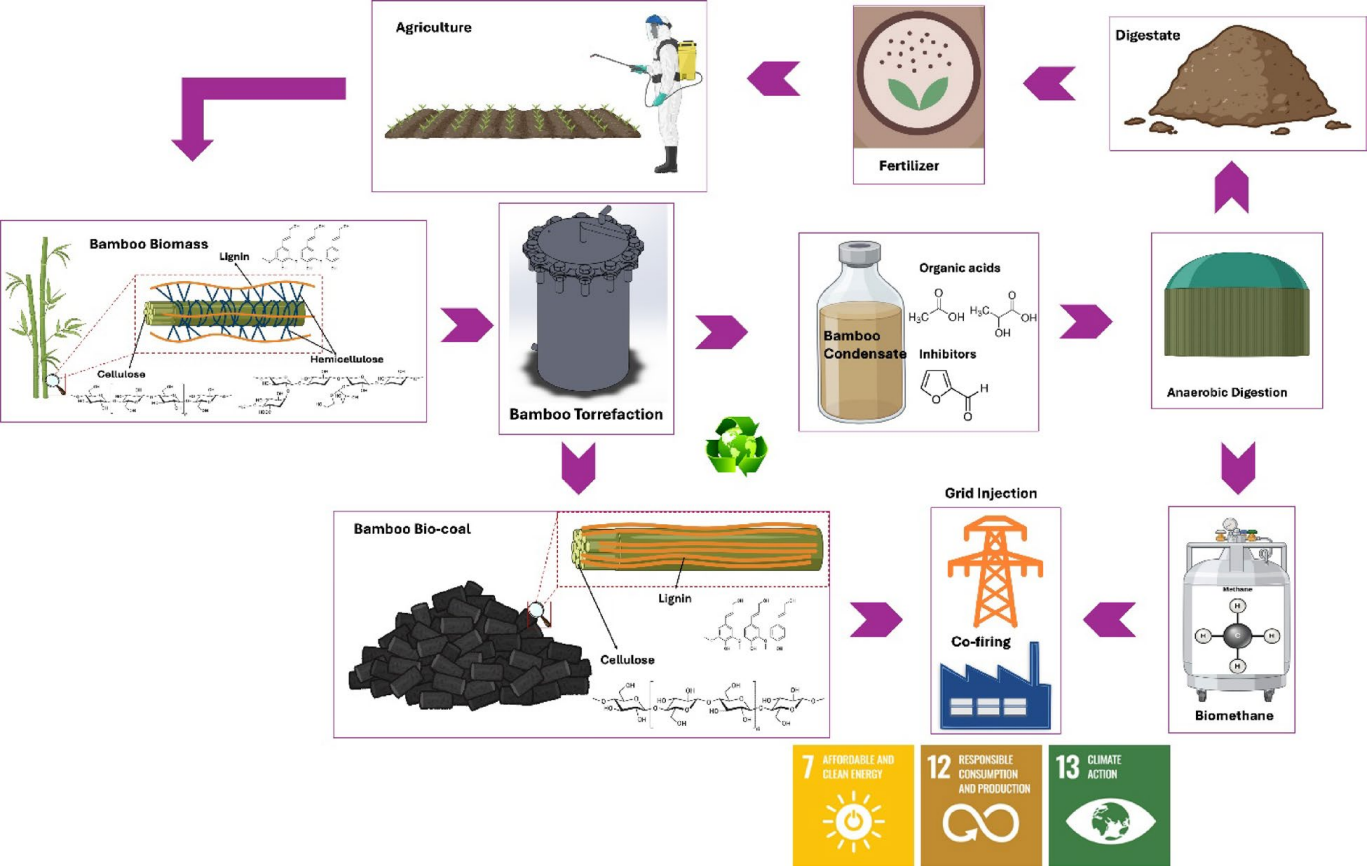
Mechanistic pathway of integrated torrefaction-anaerobic digestion of bamboo waste showing carbon densification, condensate biodegradation, and circular economy outputs.

The integrated thermochemical-biochemical pathway (Fig. 7) governs both bio-coal formation and condensate valorization. During torrefaction, hemicellulose undergoes near-complete depolymerization (99.0 $\:\pm\:$ 0.4%), accompanied by degradation (45.0 $\:\pm\:$ 2.6%) and lignin enrichment, resulting in carbon dense bio-coal fraction. In parallel, volatile degradation products condense into an aqueous phase containing biodegradable organics such as acetic acid (3.79 $\:\pm\:$ 0.02 wt%), formic acid (1.67 $\:\pm\:$ 0.05 wt%), and lactic acid (1.42 $\:\pm\:$ 0.03 wt%) (Table 1).

These intermediates play a critical role in AD, supporting both acetoclastic and hydrogenotrophic methanogenesis pathways.

Although inhibitory compounds such as furfural, phenol, and methanol were detected (Table 1), their concentrations were sufficiently low to avoid significant microbial inhibition. The digestion profile (Fig. 6) shows only minor lag-phase extension, indicating sub-inhibitory effects rather than a reduction in cumulative biomethane yield. This interpretation is based on observed BMP behavior rather than direct inhibition assays. Detailed inhibition studies are recommended for future work to quantify the impact of specific compounds on AD performance.

The high biomethane yield (493.0 $\:\pm\:$ 1.7 mL/g-VS) suggests strong biodegradability, while low standard deviations indicate high reproducibility and process stability. The nutrient-rich digestate can be reused as biofertilizer, reinforcing circular resource utilization.

### Inorganic transformation and combustion behavior

Bamboo-derived bio-coal exhibits significantly lower ash content (~ 3 wt%) compared to rice husk (~ 17 wt%) and rice straw (~ 18 wt%) (Table S5), reducing slagging and fouling tendencies during combustion. This enhances thermal efficiency, combustion stability, and operational reliability.

Furthermore, Tof-SIMS analysis (Fig. 3) reveals a relatively homogenous distribution of inorganic species in bamboo-derived bio-coal. This uniformity is attributed to enhanced mobility of metals following degradation of structural organics during torrefaction, which minimizes localized accumulation of ash-forming elements^17^.

These characteristics collectively reduce operational challenges such as clinker formation, corrosion, and maintenance requirements^7,8^.

### Comparative performance with other biomass

To benchmark performance, bamboo-derived products were compared with rice husk and rice straw under identical conditions^16^. Bamboo-derived bio-coal exhibited the highest HHV (25.4 $\:\pm\:$ 1.5 MJ/kg), outperforming rice husk (22.3 $\:\pm\:$ 0.5 MJ/kg) and rice straw (18.2 $\:\pm\:$ 0.3 MJ/kg) (Table S5). This improved performance is attributed to higher lignin content and lower ash fraction, which enhance carbon retention and reduce inert dilution.

Bamboo-derived condensate exhibited a high biomethane yield (493.0 $\:\pm\:$ 1.7 mL/g-VS), marginally lower than rice husk-derived condensate (508.0 $\:\pm\:$ 2.7 mL/g-VS) but higher than rice straw-derived condensate (471 $\:\pm\:$ 3.5 mL/g-VS). The marginal difference is likely due to higher phenolic content, while improved performance over rice straw reflects greater availability of biodegradable intermediates.

Additionally, compared to other lignocellulosic residues such as sugarcane bagasse, which typically exhibit H/C and O/C ratios of ~ 1.3–1.5 and ~ 0.5–0.6, respectively, due to their higher hemicellulose and cellulose content^19,20^, bamboo waste shows comparable initial atomic ratios, reflecting its lignocellulosic composition. In the Van Krevelen diagram (Fig. 2b), the bamboo waste in this study exhibited H/C and O/C ratios of ~ 1.40 and 1.03, respectively. With increasing torrefaction severity, both ratios decreased progressively, indicating enhanced carbonization and deoxygenation. This behavior is associated with the preferential degradation of hemicellulose and cellulose fractions and the relative enrichment of lignin, which contributes to the formation of more carbon-dense structures. These trends highlight the suitability of bamboo waste as a feedstock for integrated thermochemical-biochemical conversion systems. Overall, the results are internally consistent and align with established thermochemical conversion pathways of lignocellulosic biomass.

### Integrated energy recovery and process efficiency

With bio-coal and condensate yields of 65% and 25%, respectively, bamboo waste achieved an estimated net energy yield of 21 GJ/ton, exceeding rice husk (19 GJ/ton) and rice straw (16 GJ/ton). The integrated torrefaction and AD approach developed herein delivers a higher total energy yield (~ 21 GJ/ton), outperforming standalone torrefaction (~ 10 GJ/ton)^11^, pyrolysis (~ 13 GJ/ton)^19^, and gasification (~ 12 GJ/ton)^12^ (Table 3).

**Table 3:** Comparison of thermochemical conversion technologies applied to bamboo.

Technology	Products	Energy Yield (GJ/ton of bamboo)	Integrated Valorization	Operational Complexity	References
Standalone Torrefaction	Torrefied biomass	~ 10	No	Low- single step, low temperature	^11^
Pyrolysis	Bio-oil, Biochar, Syngas	~ 13	No	High- High temperature, thermal control & product recovery required	^19^
Gasification	Syngas	~ 12	No	High- high temperature, syngas cleanup needed	^12^
**Torrefaction + AD**	**Bio-coal**,** Bio-methane**	**~ 21**	**Yes**	**Medium- Requires integration with biological system**	**This study**

This improvement arises from process integration, wherein volatile fractions generated during torrefaction are captured and converted into methane rather than being lost, thereby maximizing carbon utilization efficiency.

This integrated approach developed here overcomes a common limitation of standalone thermochemical systems, where volatile fractions are often underutilized, thereby improving overall process efficiency and resource utilization.

### Techno-economic feasibility and scalability

The economic feasibility of the integrated torrefaction-AD system is supported by the widespread availability of bamboo waste, particularly in biomass-rich regions such as northeastern India^5^. This enables decentralized plant configurations, which reduce transportation distances and associated logistical costs while enhancing feedstock security.

The total capital investment for the 50,000 t/y facility is estimated at US$ 24.13 million, with an annual production cost of US$ 3.62 million (Table 4). The slightly higher production cost compared to rice husk/straw systems is primarily attributed to the higher feedstock price of bamboo^16,21^. The feedstock cost (US$ 39 per ton)^21^ reflects procurement at source, while transportation and size reduction (grinding costs) are incorporated within operational expenditures (utilities and handling), ensuring a realistic representation of industrial conditions.

**Table 4:** Total capital investment, production costs, and revenue for integrating bamboo torrefaction with AD (50000 t/y plant capacity)^16,22,23^.

Category	Equipment	Cost parameters	Cost (US $)
Capital Investment	Conveyor	6.3 t/h	235,685
	Blower	–	120,215
	Dryer	5 t/h x 6 units	1,320,457
	Boiler	2100 kW	375,250
	Bio-coal cooler	–	307,082
	Bio-coal screening	–	83,680
	Bio-coal storage	–	56,716
	Volatile condensor	50 t/d	112,675
	Cyclones and filters	50 t/d	142,381
	Condensate separator	–	24,301
	Torrefaction reactor	6.3 t/h, 330 days	9,397,893
	Anaerobic digester	5000 m^3^	1,805,265
	Digestate storage tanks	7 t/d	51,711
	Installation	Heating, pumps, etc.	522,391
	Biogas upgrading	High pressure water scrubbing	281,850
	Heat exchanger	Energy recovery	359,044
	Site and building	Paving, receiving station, load area, building, and office space	702,123
	Total equipment + infra	Sum of total equipment cost and site and building cost	15,898,719
	Startup expenses	10% on above total	1,589,871
	Engineering and supervision cost	12% on above total	1,907,846
	Contingency	10% on above total	1,589,871
	Working capital	15% on total + extras	3,147,946
Total capital investment		–	**24**,**134**,**253**
Production cost	**Cost item**	**Cost parameters**	**Cost (US $)**
	Feedstock	50,000 t @ US$ 39/t	1,950,000
	Utilities	–	132,500
	Maintenance	3% of total fixed capital	209,863
	Manpower	3 operators + 2 supervisors	45,710
	Factory overheads	Payroll, employee benefits, medical facilities, accounting, safety and emergency services	148,676
	Administration costs	Estimated	43,195
	Waste treatment	–	14,079
	Distribution and selling	Estimated	17,968
	Depreciation	15 years straight-line	1,059,915
Total production cost			**3**,**621**,**906**
Revenue	**Products**	**Cost parameters**	**Cost (US $)**
	Bio-coal	~ 65 wt% yield from 50,000 t/y = 32,500 t/y x US$ 69/t	2,242,500
	Bio-methane	148 m^3^/h. x US$ 1.3/m^3^	1,523,808
	Heat	1.03 MWh @ US$ 65/MWh	530,244
Net Total revenue			**4**,**296**,**552**

The integrated system generates a total annual revenue of ~ US$ 4.30 million, with bio-coal contributing the largest share due to its higher yields (~ 65 wt%) and market value (US$ 69/ton)^24^. The inclusion of AD significantly enhances economic performance by generating additional revenue from biomethane (~ US$ 1.52 million annually) and heat recovery^25,26^. Overall, process integration results in a ~ 47% increase in revenue compared to standalone torrefaction systems (Table 5).

**Table 5:** Comparative economic metrics for bamboo waste, rice husk/straw-based bio-coal production systems integrated with anerobic digestion.

Economic metric	Bamboo waste (this study)	Rice husk/straw^16^
Capital investment	US$ 24,134,253	US$ 24,134,253
Feedstock cost	US$ 1,950,000	US$ 1,300,000
Production cost	US$ 3,621,906	US$ 2,964,017
Net revenue	US$ 4,296,552	US$ 3,629,052
NPV	US$ 8,545,000	US$ 5,580,000
IRR	15.8%	11.5%
Payback period	6.5 years	7.2 years
Profitability index	1.34	1.23

The economic assumptions employed in this study are based on a combination of literature-reported values, industrial benchmarks, and market-aligned pricing^22–27^, ensuring realistic commercial scenarios. While lignin is conservatively included in the bio-coal fraction in the current techno-economic assessment, it has significant potential for conversion into high-value products such as phenolic resins, carbon materials, and platform chemicals^28,29^. Diverting lignin to these applications could substantially increase revenue and improve profitability, represents a promising avenue for future development. These pathways would require additional processing and market validation and are thus presented as a prospective enhancement rather than part of the current conservative assessment.

In this study, torrefaction was conducted using finely ground biomass (1–2 mm) under laboratory conditions to ensure uniform heat transfer and reproducible results. For techno-economic analysis, however, larger and industry-relevant particle sizes (3–10 mm) were assumed to reflect practical biomass handling and reduce the need for energy-intensive grinding. Bamboo waste streams, particularly from handicraft, incense stick, and flute manufacturing industries, are typically available in coarse fractions (> 2 mm) that can be directly processed without extensive preprocessing. This characteristic supports decentralized processing strategies, enabling conversion close to the biomass source and minimizing transportation and size reduction costs. Any minor preprocessing requirements are included within operational expenditures in the techno-economic assessment, ensuring a realistic and feasible economic evaluation.

### Economic performance indicators, sensitivity, and risk assessment

The economic performance indicators further confirm the viability of the integrated systems. The net present value (NPV) is estimated at US$ 8.54 million, with an IRR of 15.8%, and a payback period of 6.5 years, all of which are higher than rice husk/straw-based systems (Table 5). These results indicate the potential advantages of bamboo as a feedstock in integrated bioenergy systems.

To evaluate the robustness of these outcomes, a deterministic sensitivity analysis was conducted by varying key economic parameters, including feedstock cost, bio-coal selling price, and biomethane revenue within a $$\:\pm\:$$ 20% range (Table 6). The analysis reveals that bio-coal selling price is the most influential parameter, followed by feedstock cost, while biomethane revenue exhibits moderate sensitivity. Specifically, a $$\:\pm\:$$ 20% variation in bio-coal price results in an approximately $$\:\pm\:$$ 14% change in net annual profit, whereas similar variations in feedstock cost led to an ~ $$\:\pm\:$$ 11–12% change. In contrast, biomethane revenue variations result in comparatively smaller changes ($$\:\pm\:$$ 8%), although they remain important for overall process integration benefits. Sensitivity analysis suggests that inclusion of moderate transport cost (10–15 US$ per ton of biomass) does not significantly alter economic viability, although site-specific logistics may influence.

**Table 6:** Sensitivity analysis of key economic parameters on net annual profit.

Parameter	Variation	Impact on net annual profit	Interpretation
Feedstock cost	−20%	+ 11.5%	Strong positive impact due to high contribution to OPEX
	−10%	+ 5.8%	
	+ 10%	−5.8%	
	+ 20%	−11.5%	
Bio-coal selling price	−20%	−14.2%	Most sensitive parameter due to dominant revenue share
	−10%	−7.1%	
	+ 10%	+ 7.1%	
	+ 20%	+ 14.2%	
Biomethane revenue	−20%	−7.8%	Moderate sensitivity; supports process integration
	−10%	−3.9%	
	+ 10%	+ 3.9%	
	+ 20%	+ 7.8%	

Importantly, despite these variations, the system maintains positive economic indicators (NPV > 0 and IRR > 10%) under moderate fluctuations, indicating strong economic resilience.

A preliminary risk assessment further identifies key uncertainties associated with feedstock price variability, regional logistics, and market dynamics. However, the decentralized nature of the torrefaction process, combined with the use of locally available bamboo waste, inherently mitigates supply chain risks. Moreover, the integrated production of multiple energy products (bio-coal, biomethane, and heat) distributes revenue streams across different markets, thereby reducing dependence on a single product and enhancing overall financial stability.

Overall, the results demonstrate that the combination of decentralized processing, favorable feedstock characteristics, and process integration not only improves economic performance but also enhances system robustness under real-world operating conditions.

### Limitations, future perspectives, and circular bioeconomy implications

While this study provides a comprehensive experimental and techno-economic assessment of bamboo waste torrefaction integrated AD, the following limitations should be considered. The techno-economic analysis is based on a decentralized plant configuration, and transport costs were considered minimal due to localized biomass sourcing; however, regional variability in logistics could influence overall feasibility. The BMP experiments were conducted using a single, literature-supported substrate-to-inoculum ratio (VS_substrate_:VS_inoculum_), and variations in substrate-to-inoculum ratio may influence digestion kinetics and methane yield. Additionally, inhibition effects during AD were not explicitly quantified and warrant further investigation. Kinetic modeling (e.g., modified Gompertz model) of AD process was beyond the scope of this work and represents an important direction for future research. Furthermore, a detailed life cycle assessment would be valuable to fully evaluate environmental impacts. These limitations do not affect the validity of the comparative framework but highlight opportunities for further process optimization and scale-up validation.

Nonetheless, the integrated system indicates clear potential for advancing sustainable bioenergy production. By valorizing both solid and liquid fractions and recycling digestate as fertilizer, the system achieves near-complete resource utilization, supporting Sustainable Development Goals 7, 12, and 13. These findings highlight the role of such integrated approaches in promoting circular bioeconomy principles.

In summary, bamboo waste emerges as a promising feedstock for integrated bioenergy production. By combining mechanistic understanding with techno-economic validation, this study establishes that bamboo offers synergistic advantages in fuel quality, energy recovery, and economic performance, supporting its deployment in circular bioeconomy strategies, particularly in bamboo-rich regions.

## Conclusion

This study demonstrates the feasibility of an integrated torrefaction–anaerobic digestion pathway for the near-complete valorization of bamboo waste, enabling simultaneous recovery of energy from both solid and liquid streams. Torrefaction produced low-ash, carbon-enriched bio-coal (HHV: 25.4 MJ/kg), while the condensate was effectively converted to biomethane (493 mL CH₄/g VS), transforming an underutilized by-product into a valuable energy resource. The integrated process achieved a net energy recovery of ~ 21 GJ/ton, exceeding rice husk and rice straw under identical conditions within a controlled comparative framework. Techno-economic analysis indicated favourable economic performance (IRR: 15.8%, NPV: US$ 8.54 million), based on experimentally derived mass and energy balances. Overall, this study provides a consistent and experimentally validated framework for integrated, feedstock-specific bioenergy systems with potential relevance for decentralized applications in biomass-rich regions.

## Materials and methods

### Feedstock

The bamboo waste was procured from Khanpur bamboo market, New Delhi, India. The collected bamboo chips were rinsed with distilled water to remove dust. Later, the feedstock was dried at 105 °C for 24 h, finely ground, and sieved to a particle size of 1–2 mm for analysis and batch torrefaction experiments. While 1–2 mm particles were used for laboratory-scale experiments to maintain experimental uniformity, larger particle sizes (3–10 mm) were assumed for industrial-scale scenarios to align experimental findings with real-world feasibility. Detailed characterization techniques are described in Kachroo et al.^17^.

### Torrefaction setup and procedure

**Fig. 8:**
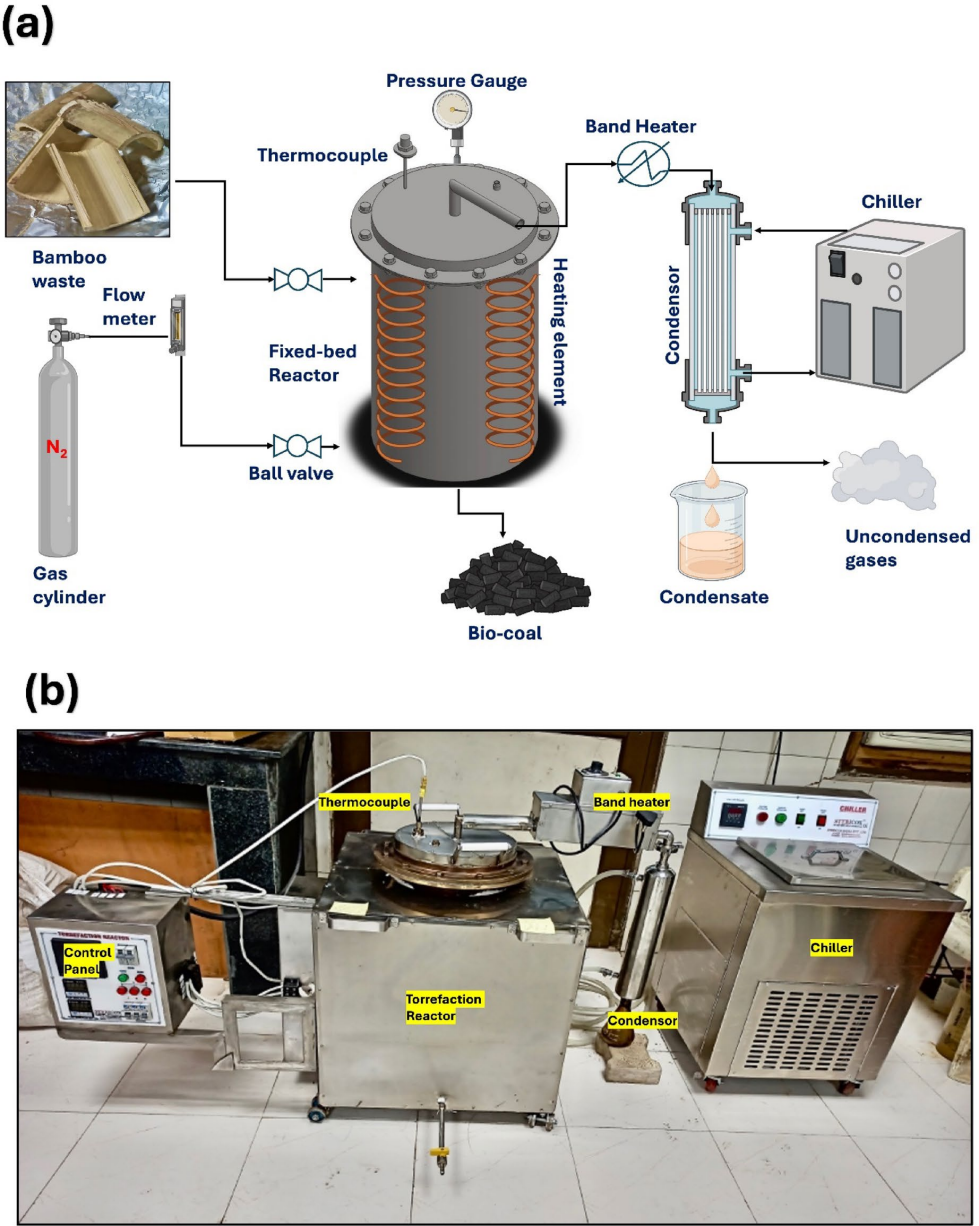
(**a**) Schematic diagram of fixed-bed torrefaction reactor unit. (**b**) Experimental setup of fixed-bed torrefaction reactor unit (Reproduced from Kachroo et al.^16^ with permission).

Bamboo waste torrefaction was conducted in a lab-scale fixed-bed thermochemical reactor coupled with a counter-flow tube-type condenser for volatile condensation (Fig. 8a). The SS316 torrefaction reactor design (Fig. 8b) is detailed in our previous study^16^. Torrefaction was conducted in a controlled environment with an inert N_2_ flow (100 mL/min) maintained to aid volatile removal, and reactor temperatures were monitored with a thermocouple and maintained within $\:\pm\:$ 5 °C using a PID controller. A band heater near the condenser inlet prevented premature condensation of volatile vapors in the outlet pipe (Fig. 8a). These vapors condensed into torrefaction condensate. At the same time, uncondensed gases were collected in 500 mL Tedlar bags at the condenser outlet.

For each batch run, 1 kg of dried, finely ground (1–2 mm) bamboo waste was loaded into the reactor. Torrefaction was conducted at 215, 230, 245, 260, 275 and 290 °C for 30, 60, and 90 min. The temperature was ramped at 5 and 10 °C/min from room temperature (~ 30 °C) to the desired temperature. Vapors passed through a heated stainless-steel pipe (250–300 °C) to the condenser, which was maintained at ~ 1 °C using a chiller. The collected condensate was stored at 4 °C to prevent ageing. The uncondensed gases were collected for compositional analysis.

The torrefaction product and by-product yields were calculated on a mass basis, and the energy yields were estimated by the Eqn reported by Kachroo et al.^16^.

### Mass and energy distribution

The mass distribution of bamboo waste in the fixed-bed torrefaction reactor was carried out as expressed in Eq. (1).

1 $$\:{m}_{bamboo}\:=\:{m}_{biocoal\:}+\:{m}_{condensate}+\:{m}_{gas}$$

Whereas m_bamboo_ is mass of bamboo waste (Kg), m_biocoal_ is mass of bio-coal (Kg), m_condensate_ is mass of torrefaction condensate (Kg), and m_gas_ is mass of uncondensed volatiles (Kg).

The energy distribution of the bamboo torrefaction process is described in Eq. (2).

2 $$\:{E}_{bamboo}\:=\:{E}_{biocoal\:}+\:{E}_{condensate}+\:{E}_{gas}$$

Where the input energy of bamboo waste is E_bamboo_ (MJ), and the energy generated by bio-coal, torrefaction condensate, and uncondensed volatiles is E_biocoal_, E_condensate_, and E_gas_ (MJ), respectively. The reference condition values for this study were a temperature of T_i_ = 25 °C and a pressure of P_0_ =1 atm.

Energy calculations for each fraction were performed using the measured masses and HHVs as per the following Eqns.:

The input energy of the feedstock (MJ) was determined using Eq. (3).

3 $$\:{E}_{bamboo}\:=\:{m}_{bamboo}\:\times\:\:{HHV}_{bamboo}$$

Where m_bamboo_ denotes the mass of bamboo charged into the torrefaction reactor (Kg), and HHV_bamboo_ is the HHV of bamboo (MJ/kg).

The energy of bio-coal, torrefaction condensate, and uncondensed volatiles were calculated by Eqs. (4, 5, and 6).

4 $$\:{E}_{bio-coal}\:=\:{m}_{bio-coal}\:\times\:\:{HHV}_{bio-coal}$$

5 $$\:{E}_{condensate}\:=\:{m}_{condensate}\:\times\:\:{HHV}_{condensate}+\:{m}_{condensate}\:\times\:\:{Cp}_{condensate}\:\times\:({T}_{O}-\:{T}_{i})$$

6 $$\:{E}_{gas}\:=\:{m}_{gas}\:\times\:\:{HHV}_{gas}$$

7 $$\:{HHV}_{gas}\:=\left({HHV}_{{H}_{2}}\times\:\:{H}_{2}\%\right)+\left({HHV}_{CO}\times\:CO\%\right)+({HHV}_{{CH}_{4}}\times\:\:{CH}_{4}\%)$$

Where the heat capacity of condensate and inert gas were considered as 0.0024 and 0.00104 MJ/kg-K^1^, the HHV of uncondensed volatiles was calculated using Eq. (7), where the HHV of H_2_, CO, and CH_4_ was 141.8, 10.1, and 55.5 MJ/kg^16^. T_O_ denotes the operational torrefaction temperature (K).

### BMP assay

The bamboo-derived torrefaction condensate was subjected to batch BMP assays in serum bottles (120 mL) with a working volume of 60 mL at 35 °C (mesophilic conditions). Inoculum was mesophilic digester sludge (stored at 4 °C) from the Waste Treatment Lab, Department of Biochemical Engineering and Biotechnology, Indian Institute of Technology Delhi. Before incubation, the inoculum was acclimatized to mesophilic conditions for 2 days in the electric oven. The bamboo-derived torrefaction condensate, comprising 69 wt% water and tar-like viscous carbon substances, was separated using a separatory funnel. Due to the significant differences in densities, the mixture naturally formed two phases: the heavier, viscous tar phase settled at the bottom, while the aqueous condensate remained at the top. The tar phase was carefully drained, leaving the aqueous fraction intact. This step ensured efficient removal of inhibitory tar-like compounds prior to AD. The aqueous condensate was further filtered through Whatman No.1 filter paper to remove residual particulates, pH-adjusted to 7.0 using 1 M NaOH to minimize microbial inhibition, stored at 4 °C, and used within 24 h to maintain the stability of volatile fatty acids. The torrefaction condensate (after removing tar) was mixed with inoculums at VS_substrate_:VS_inoculum_ ratios of 0.1. The ratio was selected based on established literature, including our previous work^16^ and Doddapaneni et al.^13^, to ensure stable digestion conditions, sufficient buffering capacity, and reproducible methane yields while minimizing the risk of acidification or inhibition. Blanks containing only water and inoculum were used to account for background methane. Bio-methane yields were corrected for baseline methane by subtracting values from inoculum-only blanks. All experiments were performed in triplicate, and results are reported as mean and standard deviation, ensuring a thorough data analysis.

### Analytical techniques

Bio-coal, torrefaction condensate and uncondensed gases from bamboo waste torrefaction were characterized. Proximate analysis (moisture, ash, volatile matter, and fixed carbon), HHV, and elemental analysis (CHNSO) of bio-coals were performed following American Society for Testing and Materials standards D3173, D3175, E1755, D2015-96, and D5373-21^16^. Crude fibre content (hemicellulose, cellulose, and lignin) was analyzed using the Van Soest method^17^. All calculations, including atomic ratios, were independently verified to ensure internal consistency. TGA analysis and Tof-SIMS measurements were conducted per previously described protocols and instrumentation^17^. Tof-SIMS analysis was conducted to examine the spatial distribution and migration of inorganic species (e.g., K, Na, Ca) during torrefaction, providing insights into ash transformation and fuel quality.

Condensate composition was analyzed using a GC-MS system (Agilent series 7010B with 7890B MS and HP-5MS column: 30 m, 0.25 mm ID, 0.25 μm film)^16^. The chemical oxygen demand (COD, APHA 5220D), volatile solids (APHA 2540), and total solids were determined via standard protocols^13^, with soluble COD analyzed post-filtration (0.45 μm membrane filter). pH was measured using a calibrated pH meter, and water content by Karl Fischer titration.

Uncondensed gas composition and bio-methane concentration were determined using a GC (NUCON 5700) with a TCD detector and Shin Carbon ST 100/1200 micro-packed column (2 m, 1 mm ID, 1.5 mm OD)^16^. Gas analysis was supported by dedicated software. Bio-methane concentrations were benchmarked against calibration mixtures (N_2_: 40%, CH_4_: 30%, and CO_2_: 30%) and reported at standard temperature and pressure (STP).

All experiments and analytical measurements were conducted in triplicate (*n* = 3), and the results are presented as mean ± standard deviation, with the ± values reflecting experimental reproducibility and inherent measurement variability.

### Techno-economic assessment

Capital investment for bamboo torrefaction was estimated by scaling up the process to a 50,000 t/y plant capacity using mass and energy balances^28–34^. Equipment costs (torrefaction reactor, condensation units, heat exchangers, digesters, and boilers) were obtained from literature^16,28–34^ and updated to current-year equipment costs via chemical engineering plant cost index formula. Fixed capital included all equipment costs and additional expenditures (engineering, startup, supervision, and contingency)^28–34^.

Working capital was estimated at 15% to the fixed capital. Total capital investment was calculated by aggregating working and fixed capital, based on continuous torrefaction-AD plant operation (24 h/day, 330 days/year, i.e. 7920 h/year across three 8-hour shifts). The pilot-scale system was assumed to operate as a decentralized unit located near the biomass generation site, thereby minimizing transportation requirements. Any minor transportation and handling involved (e.g., short-distance movement within or between nearby industrial facilities) were considered negligible relative to other operational costs like feedstock procurement, utilities, manpower, etc., and were implicitly included within the overall operating expenses. Furthermore, the bamboo waste obtained from handicraft, incense stick, and flute manufacturing industries is typically available in the form of residues in the particle sizes in the range of 1–10 mm, which is suitable for direct torrefaction. Therefore, no additional grinding or size reduction was required, and these costs were not included as separate components in the techno-economic assessment. This assumption is consistent with decentralized biomass processing systems, where feedstock logistics and preprocessing requirements are inherently minimized. A preliminary assessment indicates that inclusion of minor transportation or preprocessing costs would have a negligible impact on the overall production cost and does not significantly alter the techno-economic conclusions.

Utility and operational costs were referenced from Kachroo et al.^16^, and workforce requirements followed the methodology of Doddapaneni et al.^22^. Supervision costs were calculated as 15% of operating labor expenses, while maintenance/repair costs were estimated at 3% of total fixed capital. Additional operating costs, including administrative, sales, distribution and factory overheads (safety services, medical, accounting, employee benefits, and payroll), were estimated using established correlations^16,22^. Depreciation was calculated using the straight-line method over 15 years. Assumptions were selected based on literature benchmarks and conservative estimates to avoid overprediction of system performance^28–34^.

The IRR, NPV, and other financial metrics were calculated to determine the techno-economic assessment of the integrated bamboo waste torrefaction-AD pilot plant.

## Supplementary Information

Below is the link to the electronic supplementary material.

Supplementary Material 1

## Data Availability

The datasets generated and analyzed during the current study are available from the corresponding author on reasonable request.

## Abbreviations

AD: Anaerobic digestion
BMP: Bio-methane potential
COD: Chemical oxygen demand
GC: Gas chromatography
HHV: Higher heating value
IRR: Internal rate of return
MS: Mass spectrometry
NPV: Net present value
SDG: Sustainable development goals
TCD: Thermal conductivity detector
TGA: Thermogravimetric analysis
Tof-SIMS: Time of flight-secondary ion mass spectrometry
VS: Volatile solids

## References

[1] Baghel, P., Sakhiya, A. K. & Kaushal, P. Influence of temperature on slow pyrolysis of Prosopis Juliflora: An experimental and thermodynamic approach. *Renew. Energy*. **185**, 538–551. 10.1016/j.renene.2021.12.053 (2022).

[2] Supraja, K. V. et al. Biochar production and its environmental applications: Recent developments and machine learning insights. *Bioresour Technol.* **387**, 129634. 10.1016/j.biortech.2023.129634 (2023). 37573981 10.1016/j.biortech.2023.129634

[3] Lefebvre, D. et al. Biomass residue to carbon dioxide removal: quantifying the global impact of biochar. *Biochar* **5**, 65. 10.1007/s42773-023-00258-2 (2023).

[4] Kumar, A. et al. Multifaceted applications of biochar in environmental management: a bibliometric profile. *Biochar* **5**, 11. 10.1007/s42773-023-00207-z (2023).

[5] National Bamboo Mission, Ministry of Agriculture & Farmers Welfare, Government of India. (2022). https://nbm.da.gov.in (Accessed 15 October 2024).

[6] Raut, P., Kaple, S., Ilorkar, V., Deshmukh, A. P. & Pandiyan, K. Composting of incense industrial bamboo waste with and without added organic, inorganic and effective microorganism as a renewable alternative. *Int. J. Adv. Biochem. Res.* **8**, 798–806. 10.33545/26174693.2024.v8.i5j.1190 (2024).

[7] Werther, J., Saenger, M., Hartge, E. U., Ogada, T. & Siagi, Z. Combustion of agricultural residues. *Prog Energy Combust. Sci.* **26**, 1–27. 10.1016/S0360-1285(99)00005-2 (2000).

[8] Vamvuka, D. & Kakaras, E. Ash properties and environmental impact of various biomass and coal fuels and their blends. *Fuel Process. Technol.* **92**, 570–581. 10.1016/j.fuproc.2010.11.013 (2011).

[9] Chen, W. H. & Kuo, P. C. Torrefaction and co-torrefaction characterization of hemicellulose, cellulose and lignin as well as torrefaction of some basic constituents in biomass. *Energy* **36**, 803–811. 10.1016/j.energy.2010.12.036 (2011).

[10] Phanphanich, M. & Mani, S. Impact of torrefaction on the grindability and fuel characteristics of forest biomass. *Bioresour Technol.* **102**, 1246–1253. 10.1016/j.biortech.2010.08.028 (2011). 20801023 10.1016/j.biortech.2010.08.028

[11] Park, S. et al. Identifying fuel characteristics of bamboo chips as a solid biofuel through torrefaction. *J. Mater. Cycles Waste Manag*. **26**, 2804–2813. 10.1007/s10163-024-02002-9 (2024).

[12] Hu, J. et al. Combustions of torrefaction-pretreated bamboo forest residues: Physicochemical properties, evolved gases, and kinetic mechanisms. *Bioresour Technol.* **304**, 122960. 10.1016/j.biortech.2020.122960 (2020). 32062500 10.1016/j.biortech.2020.122960

[13] Doddapaneni, T. R. K. C. et al. Anaerobic batch conversion of pine wood torrefaction condensate. *Bioresour Technol.* **225**, 299–307. 10.1016/j.biortech.2016.11.073 (2017). 27898321 10.1016/j.biortech.2016.11.073

[14] Fagernäs, L., Kuoppala, E., Arpiainen, V. & Composition Utilization and Economic Assessment of Torrefaction Condensates. *Energy Fuels*. **29**, 3134–3142. 10.1021/acs.energyfuels.5b00004 (2015).

[15] Chen, Y-C., Liu, A-C. & Chou, C-Y. Anaerobic Co-digestion of Bamboo Biomass and Swine Manure In: 2017 Spokane, Washington July 16 - July 19, 2017. American Society of Agricultural and Biological Engineers, St. Joseph, MI. doi:10.13031/aim.201700932 (2017).

[16] Kachroo, H. et al. Integrated biofuel production from rice husk and rice straw via torrefaction and anaerobic digestion: Optimization and economic viability. *Energy Convers. Manag*. **340**, 119952. 10.1016/j.enconman.2025.119952 (2025).

[17] Kachroo, H., Verma, V. K., Doddapaneni, T. R. K. C., Kaushal, P. & Jain, R. Organic/metallic component analysis of lignocellulosic biomass: A thermochemical-perspective-based study on rice and bamboo waste. *Bioresour Technol.* **403**, 130835. 10.1016/j.biortech.2024.130835 (2024). 38750827 10.1016/j.biortech.2024.130835

[18] Chen, W. H., Liu, S. H., Juang, T. T., Tsai, C. M. & Zhuang, Y. Q. Characterization of solid and liquid products from bamboo torrefaction. *Appl. Energy*. **160**, 829–835. 10.1016/j.apenergy.2015.03.022 (2015).

[19] Shezi, M. & Kiambi, S. L. Isothermal Pyrolysis of Bamboo and Pinewood Biomass: Product Characterization and Comparative Study in a Fluidized Bed Reactor. *Bioengineering* **12**, 99. 10.3390/bioengineering12020099 (2025). 40001620 10.3390/bioengineering12020099 PMC11852079

[20] Pérez, N. P. et al. Unlocking the potential of sugarcane bagasse: a comprehensive analysis for advanced energy conversion. *Bioresour Bioprocess.* **12**, 60. 10.1186/s40643-025-00878-5 (2025). 40524102 10.1186/s40643-025-00878-5 PMC12170471

[21] Aayog, N. I. T. I. Ministry of Agriculture & Farmers Welfare, Government of India Road map of round bamboo sticks (2022). Available at: https://www.niti.gov.in/sites/default/files/2022-02/Bamboo_Presentations/Technical_Session_4_Roadmap_of_Round_Bamboo_Stick_Market_Shri_Pratap_Goswami.pdf (Accessed 10 March 2025).

[22] Doddapaneni, T. R. K. C. & Kikas, T. Integrating torrefaction of pulp industry sludge with anaerobic digestion to produce bioenergy and biochemicals: Techno-economic and environmental feasibility analysis. *Chem. Eng. J. Adv.* **14**, 100463. 10.1016/j.ceja.2023.100463 (2023).

[23] Doddapaneni, T. R. K. C., Praveenkumar, R., Tolvanen, H., Rintala, J. & Konttinen, J. Techno-economic evaluation of integrating torrefaction with anaerobic digestion. *Appl. Energy*. **213**, 272–284. 10.1016/j.apenergy.2018.01.045 (2018).

[24] IndiaMART Biomass briquettes product listing IndiaMART. (2023). Available at: https://export.indiamart.com/products/?id=2849536989712&pos=15&kwd=biomass%20briquettes&tags=A (Accessed 18 June 2025).

[25] Purchase price of compressed bio gas (CBG) under SATAT scheme. (2023). https://satat.co.in/satat/assets/download/CBG%20Pricing%20Circular%20-%20Stakeholders.pdf (Accessed 08 October 2024).

[26] Thermal electricity storage in India retrofitting potential for coal-fired power plants in India. (2022). https://energyforum.in/fileadmin/india/media_elements/publications/20230418_Carnot_Battery/20230418_mn_Carn.pdf (Accessed 28 October 2024).

[27] Doddapaneni, T. R. K. C. et al. Adsorption of furfural from torrefaction condensate using torrefied biomass. *Chem. Eng. J.* **334**, 558–568. 10.1016/j.cej.2017.10.053 (2018).

[28] Yuan, Z., Bals, B. D., Hegg, E. L. & Hodge, D. B. Technoeconomic evaluation of recent process improvements in production of sugar and high-value lignin co-products via two-stage Cu-catalyzed alkaline-oxidative pretreatment. *Biotechnol. Biofuels Bioprod.* **15**, 45. 10.1186/s13068-022-02139-5 (2022). 35509012 10.1186/s13068-022-02139-5 PMC9069716

[29] Barbera, E., Guarise, A., Bertucco, A., Maglinao, R. L. & Kumar, S. Lignin valorization in biorefineries: A techno-economic analysis of a novel process for biolubricant production from lignin and waste cooking oil. *J. Supercrit Fluids*. **223**, 106631. 10.1016/j.supflu.2025.106631 (2025).

[30] Akbari, M., Oyedun, A. O. & Kumar, A. Techno-economic assessment of wet and dry torrefaction of biomass feedstock. *Energy* **207**, 118287. 10.1016/j.energy.2020.118287 (2020).

[31] Osman, A. I. et al. Life cycle assessment and techno-economic analysis of sustainable bioenergy production: a review. *Environ. Chem. Lett.* **22**, 1115–1154. 10.1007/s10311-023-01694-z (2024).

[32] Cahyanti, M. N., Doddapaneni, T. R. K. C. & Kikas, T. Biomass torrefaction: An overview on process parameters, economic and environmental aspects and recent advancements. *Bioresour Technol.* **301**, 122737. 10.1016/j.biortech.2020.122737 (2020). 31982296 10.1016/j.biortech.2020.122737

[33] Abelha, P. & Kiel, J. Techno-economic assessment of biomass upgrading by washing and torrefaction. *Biomass Bioenergy*. **142**, 105751. 10.1016/j.biombioe.2020.105751 (2020).

[34] Sarker, T. R., German, C. S., Borugadda, V. B., Meda, V. & Dalai, A. K. Techno-economic analysis of torrefied fuel pellet production from agricultural residue via integrated torrefaction and pelletization process. *Heliyon* **9**, e16359. 10.1016/j.heliyon.2023.e16359 (2023). 37260899 10.1016/j.heliyon.2023.e16359 PMC10227336

