# Supplementary material for: Integrated torrefaction-anaerobic digestion of bamboo waste for enhanced energy recovery: process optimization, product characterization, and techno-economic evaluation
Source: Sci Rep. 2026 May 21;16:15878. doi: 10.1038/s41598-026-52760-9 (PMC13194923; doi:10.1038/s41598-026-52760-9)
Supplement: Supplementary file 1 — Supplementary Material 1 [file 41598_2026_52760_MOESM1_ESM.docx]

**Integrated Torrefaction-Anaerobic Digestion of Bamboo Waste for Enhanced Energy Recovery: Process Optimization, Product Characterization, and Techno-Economic Evaluation**

Himanshu Kachroo ^a, b^, Tharaka Rama Krishna C. Doddapaneni ^c^, Priyanka Kaushal ^d^, Sabine Kutschke ^b^, Rohan Jain ^b*^

^a^ School of Interdisciplinary Research, Indian Institute of Technology Delhi, Hauz Khas, New Delhi-110016, India

^b^ Helmholtz Institute Freiberg for Resource Technology, Helmholtz-Zentrum Dresden-Rossendorf, Bautzner Landstraße 400, 01328, Dresden, Germany

^c^ Renewable and Sustainable Energy Research Center, Technology Innovation Institute, Masdar City, Abu Dhabi, United Arab Emirates

^d^ Clean Energy Laboratory, Centre for Rural Development and Technology, Indian Institute of Technology Delhi, Hauz-Khas, New Delhi – 110016, India

^*^ Corresponding author

Dr. Rohan Jain

Helmholtz-Zentrum Dresden-Rossendorf,

Helmholtz Institute Freiberg for Resource Technology,

Bautzner Landstraße 400,

01328, Dresden, Germany

E-Mail: [r.jain@hzdr.de](mailto:r.jain@hzdr.de), [rohanjain.iitd@gmail.com](mailto:rohanjain.iitd@gmail.com)

Phone no: - 00 49 351 260 2725

**Supplementary information**

**2. Results**

**2.3. Torrefaction product characterization at best torrefaction conditions**

**2.3.1. Bamboo-derived bio-coal characteristics**

**Table S1. Effect of torrefaction parameters on fuel characteristics and composition of bamboo.**

| **Temperature (°C)** | **Residence time (min)** | **Heating rate (°C/min)** | **Volatile matter (wt.%)** | **H/C** | **O/C** | **Hemicellulose (%)** | **Cellulose (%)** | **Lignin (%)** |
| --- | --- | --- | --- | --- | --- | --- | --- | --- |
| 215 | 30 | 5 | 65.0 $\pm$ 0.3 | 1.390 | 0.951 | 20.0 $\pm$ 0.5 | 50.0 $\pm$ 0.5 | 25.0 $\pm$ 0.3 |
| 230 | 30 | 5 | 61.0 $\pm$ 0.7 | 1.375 | 0.878 | 17.0 $\pm$ 0.8 | 51.0 $\pm$ 0.2 | 26.0 $\pm$ 0.2 |
| 245 | 30 | 5 | 56.0 $\pm$ 0.8 | 1.324 | 0.763 | 13.0 $\pm$ 0.5 | 52.0 $\pm$ 0.9 | 28.0 $\pm$ 0.3 |
| 260 | 30 | 5 | 49.0 $\pm$ 0.4 | 1.269 | 0.692 | 7.0 $\pm$ 0.8 | 50.0 $\pm$ 0.5 | 33.0 $\pm$ 0.9 |
| 275 | 30 | 5 | 39.0 $\pm$ 0.2 | 1.208 | 0.576 | 3.0 $\pm$ 0.2 | 36.0 $\pm$ 0.5 | 45.0 $\pm$ 0.4 |
| 290 | 30 | 5 | 33.0 $\pm$ 0.4 | 1.174 | 0.518 | 1.0 $\pm$ 0.6 | 28.0 $\pm$ 0.5 | 50.0 $\pm$ 0.8 |
| 215 | 30 | 10 | 64.0 $\pm$ 0.2 | 1.387 | 0.937 | 20.0 $\pm$ 0.7 | 50.0 $\pm$ 0.9 | 25.0 $\pm$ 0.6 |
| 230 | 30 | 10 | 60.0 $\pm$ 0.7 | 1.375 | 0.862 | 17.0 $\pm$ 0.2 | 51.0 $\pm$ 0.2 | 26.0 $\pm$ 0.1 |
| 245 | 30 | 10 | 55.0 $\pm$ 0.8 | 1.322 | 0.759 | 12.0 $\pm$ 0.8 | 52.0 $\pm$ 0.5 | 29.0 $\pm$ 0.7 |
| 260 | 30 | 10 | 48.0 $\pm$ 0.6 | 1.257 | 0.622 | 7.0 $\pm$ 0.5 | 50.0 $\pm$ 0.9 | 33.0 $\pm$ 0.8 |
| 275 | 30 | 10 | 38.0 $\pm$ 0.7 | 1.203 | 0.522 | 2.0 $\pm$ 0.9 | 35.0 $\pm$ 0.5 | 47.0 $\pm$ 0.6 |
| 290 | 30 | 10 | 32.0 $\pm$ 0.8 | 1.161 | 0.487 | 1.0 $\pm$ 0.2 | 27.0 $\pm$ 0.5 | 51.0 $\pm$ 0.5 |
| 215 | 60 | 5 | 63.0 $\pm$ 0.3 | 1.384 | 0.914 | 19.0 $\pm$ 0.4 | 51.0 $\pm$ 0.3 | 26.0 $\pm$ 0.8 |
| 230 | 60 | 5 | 59.0 $\pm$ 0.8 | 1.359 | 0.846 | 16.0 $\pm$ 0.3 | 51.0 $\pm$ 0.7 | 27.0 $\pm$ 0.1 |
| 245 | 60 | 5 | 54.0 $\pm$ 0.7 | 1.312 | 0.745 | 12.0 $\pm$ 0.7 | 52.0 $\pm$ 0.4 | 29.0 $\pm$ 0.8 |
| 260 | 60 | 5 | 47.0 $\pm$ 0.4 | 1.218 | 0.625 | 6.0 $\pm$ 0.4 | 51.0 $\pm$ 0.7 | 34.0 $\pm$ 0.4 |
| 275 | 60 | 5 | 37.0 $\pm$ 0.5 | 1.202 | 0.514 | 2.0 $\pm$ 0.5 | 34.0 $\pm$ 0.5 | 48.0 $\pm$ 0.3 |
| 290 | 60 | 5 | 30.0 $\pm$ 0.2 | 1.148 | 0.432 | 0.8 $\pm$ 0.1 | 26.0 $\pm$ 0.1 | 52.0 $\pm$ 0.9 |
| 215 | 60 | 10 | 62.0 $\pm$ 0.6 | 1.377 | 0.904 | 18.0 $\pm$ 0.8 | 51.0 $\pm$ 0.6 | 26.0 $\pm$ 0.7 |
| 230 | 60 | 10 | 58.0 $\pm$ 0.7 | 1.347 | 0.831 | 16.0 $\pm$ 0.1 | 52.0 $\pm$ 0.9 | 27.0 $\pm$ 0.2 |
| 245 | 60 | 10 | 53.0 $\pm$ 0.2 | 1.309 | 0.725 | 11.0 $\pm$ 0.5 | 52.0 $\pm$ 0.7 | 30.0 $\pm$ 0.3 |
| 260 | 60 | 10 | 46.0 $\pm$ 0.4 | 1.208 | 0.613 | 6.0 $\pm$ 0.2 | 51.0 $\pm$ 0.5 | 35.0 $\pm$ 0.4 |
| 275 | 60 | 10 | 35.0 $\pm$ 0.2 | 1.073 | 0.491 | 2.0 $\pm$ 0.8 | 33.0 $\pm$ 0.7 | 50.0 $\pm$ 0.9 |
| 290 | 60 | 10 | 25.0 $\pm$ 0.8 | 0.982 | 0.391 | 0.4 $\pm$ 0.1 | 22.0 $\pm$ 0.3 | 53.0 $\pm$ 0.6 |
| 215 | 90 | 5 | 62.0 $\pm$ 0.9 | 1.322 | 0.896 | 18.0 $\pm$ 0.8 | 51.0 $\pm$ 0.4 | 26.0 $\pm$ 0.8 |
| 230 | 90 | 5 | 58.0 $\pm$ 0.7 | 1.296 | 0.817 | 15.0 $\pm$ 0.4 | 52.0 $\pm$ 0.1 | 28.0 $\pm$ 0.4 |
| 245 | 90 | 5 | 53.0 $\pm$ 0.1 | 1.193 | 0.716 | 11.0 $\pm$ 0.8 | 53.0 $\pm$ 0.4 | 30.0 $\pm$ 0.5 |
| 260 | 90 | 5 | 46.0 $\pm$ 0.7 | 1.127 | 0.613 | 6.0 $\pm$ 0.1 | 51.0 $\pm$ 0.7 | 35.0 $\pm$ 0.7 |
| 275 | 90 | 5 | 35.0 $\pm$ 0.1 | 1.021 | 0.485 | 2.0 $\pm$ 0.6 | 32.0 $\pm$ 0.5 | 50.0 $\pm$ 0.5 |
| 290 | 90 | 5 | 28.0 $\pm$ 0.5 | 0.988 | 0.423 | 0.5 $\pm$ 0.1 | 25.0 $\pm$ 0.5 | 54.0 $\pm$ 0.3 |
| 215 | 90 | 10 | 61.0 $\pm$ 0.8 | 1.311 | 0.875 | 18.0 $\pm$ 0.7 | 51.0 $\pm$ 0.4 | 27.0 $\pm$ 0.2 |
| 230 | 90 | 10 | 57.0 $\pm$ 0.6 | 1.268 | 0.803 | 15.0 $\pm$ 0.1 | 52.0 $\pm$ 0.5 | 28.0 $\pm$ 0.5 |
| 245 | 90 | 10 | 52.0 $\pm$ 0.9 | 1.198 | 0.708 | 10.0 $\pm$ 0.3 | 53.0 $\pm$ 0.6 | 31.0 $\pm$ 0.6 |
| 260 | 90 | 10 | 45.0 $\pm$ 0.8 | 1.105 | 0.591 | 5.0 $\pm$ 0.9 | 52.0 $\pm$ 0.7 | 36.0 $\pm$ 0.3 |
| 275 | 90 | 10 | 34.0 $\pm$ 0.2 | 0.994 | 0.478 | 1.0 $\pm$ 0.8 | 32.0 $\pm$ 0.5 | 51.0 $\pm$ 0.9 |
| 290 | 90 | 10 | 27.0 $\pm$ 0.1 | 0.966 | 0.409 | 0.3 $\pm$ 0.1 | 24.0 $\pm$ 0.5 | 55.0 $\pm$ 0.4 |

**2.4. Mass and energy distribution**

**Table S2. Mass distribution of bamboo waste at different temperatures (residence time: 60 minutes, heating rate: 10 °C/min).**

| **Temperature (°C)** | **Input (kg)** | **Total input (kg)** | **Output (kg)** | | | **Closure (%)** |
| --- | --- | --- | --- | --- | --- | --- |
|  | **Biomass** |  | **Bio-coal** | **Condensate** | **Tor-gas** |  |
| 215 | 1 | 1 | 0.82 | 0.09 | 0.05 | 96 |
| 230 | 1 | 1 | 0.75 | 0.13 | 0.08 | 96 |
| 245 | 1 | 1 | 0.69 | 0.17 | 0.09 | 95 |
| 260 | 1 | 1 | 0.65 | 0.20 | 0.11 | 96 |
| 275 | 1 | 1 | 0.61 | 0.24 | 0.13 | 98 |
| 290 | 1 | 1 | 0.58 | 0.28 | 0.13 | 99 |

**Table S3. Mass distribution of bamboo waste at different residence times (temperature: 290 °C, heating rate: 10 °C/min) .**

| **Durations (mins)** | **Input (kg)** | **Total input (kg)** | **Output (kg)** | | | **Closure (%)** |
| --- | --- | --- | --- | --- | --- | --- |
|  | **Biomass** |  | **Bio-coal** | **Condensate** | **Tor-gas** |  |
| 30 | 1 | 1 | 0.62 | 0.25 | 0.09 | 96 |
| 60 | 1 | 1 | 0.58 | 0.28 | 0.13 | 99 |
| 90 | 1 | 1 | 0.54 | 0.31 | 0.14 | 99 |

**Table S4. Energy distribution of bamboo waste at different temperatures (residence time: 60 minutes, heating rate: 10 °C/min).**

| **Temperature (°C)** | **Input (Mj)** | **Total input (Mj)** | **Output (Mj)** | | | **Closure (%)** |
| --- | --- | --- | --- | --- | --- | --- |
|  | **Biomass** |  | **Bio-coal** | **Condensate** | **Tor-gas** |  |
| 215 | 17.6 | 17.6 | 16.39 | 0.59 | 0.32 | 98 |
| 230 | 17.6 | 17.6 | 16.06 | 0.97 | 0.47 | 99 |
| 245 | 17.6 | 17.6 | 15.62 | 1.24 | 0.51 | 98 |
| 260 | 17.6 | 17.6 | 15.55 | 1.45 | 0.57 | 99 |
| 275 | 17.6 | 17.6 | 15.03 | 1.61 | 0.91 | 99 |
| 290 | 17.6 | 17.6 | 14.75 | 1.85 | 0.95 | 99 |

**Table S5. Energy distribution of bamboo waste at different residence times (temperature: 290 °C, heating rate: 10 °C/min).**

| **Duration (mins)** | **Input (Mj)** | **Total input (Mj)** | **Output (Mj)** | | | **Closure (%)** |
| --- | --- | --- | --- | --- | --- | --- |
|  | **Biomass** |  | **Bio-coal** | **Condensate** | **Tor-gas** |  |
| 30 | 17.6 | 17.6 | 14.94 | 1.62 | 0.89 | 99 |
| 60 | 17.6 | 17.6 | 14.75 | 1.85 | 0.95 | 99 |
| 90 | 17.6 | 17.6 | 14.07 | 2.25 | 1.12 | 99 |

**3. Discussion**

This comparison reinforces that metal migration and ash composition are critical parameters influenced by feedstock type, with direct implications for operational stability and maintenance in commercial bio-coal applications. Bamboo’s performance suggests it can offer advantages in industrial torrefaction-AD systems where fouling and slagging are limiting factors.

**Table S6: Comparison of torrefaction characteristics of rice husk, rice straw, and bamboo waste: bio-coal and condensate properties**

| **Parameter** | **Rice husk [18]** | **Rice straw [18]** | **Bamboo waste** |
| --- | --- | --- | --- |
| Bio-coal moisture reduction | ~92% | ~92% | ~92% |
| Bio-coal volatile matter reduction | ~48% | ~51% | ~66% |
| Bio-coal fixed carbon increase | 12.0 $\pm$ 0.3% to 36.0 $\pm$ 0.5% | 8.0 $\pm$ 0.2% to 28.0 $\pm$ 0.3% | 16.0 $\pm$ 0.4% to 30.0 $\pm$ 0.6% |
| Bio-coal ash content increase | 14.0 $\pm$ 0.4% to 17.0 $\pm$ 0.5% | 16.0 $\pm$ 0.5% to 18.0 $\pm$ 0.2% | 2.0 $\pm$ 0.2% to 3.0 $\pm$ 0.4% |
| Bio-coal HHV increment | 16.0 $\pm$ 0.1 to 22.3 $\pm$ 0.5 MJ/kg | 13.7 $\pm$ 0.1 to 18.2 $\pm$ 0.3 MJ/kg | 17.6 $\pm$ 0.4 MJ/kg to 25.4 $\pm$ 1.5 MJ/kg |
| Bio-coal thermal decomposition peak | Higher than untreated feedstocks (~320 °C) | Higher than untreated feedstocks (~320 °C) | Higher than untreated bamboo (~315 °C) |
| Bio-coal atomic carbon increase | ~36% | ~34% | ~38% |
| Bio-coal atomic oxygen decrement | ~44%` | ~37% | ~53% |
| Hemicellulose reduction | 98.0 $\pm$ 0.3% | 80.0 $\pm$ 0.6% | 99.0 $\pm$ 0.4% |
| Cellulose reduction | 37.0 $\pm$ 0.5% | 32.0 $\pm$ 0.8% | 45.0 $\pm$ 2.6% |
| Elemental distribution (Tof-SIMS) | Homogenous | Heterogenous | Homogenous across the bulk |
| Condensate pH | 2.5 $\pm$ 0.3 | 2.7 $\pm$ 0.2 | 2 $\pm$ 0.2 |
| Condensate HHV | 10.8 $\pm$ 0.5 MJ/kg | 9.9 $\pm$ 0.4 MJ/kg | 11.4 $\pm$ 0.3 MJ/kg |
| Condensate organic acids concentration | Acetic acid: 4.0 $\pm$ 0.18 wt%, Lactic acid: 1.6 $\pm$ 0.11 wt%, Formic acid: 1.6 $\pm$ 0.03 wt% | Acetic acid: 3.5 $\pm$ 0.02 wt%, Lactic acid: 1.1 $\pm$ 0.06 wt%, Formic acid: 1.9 $\pm$ 0.02 wt% | Acetic acid: 3.8 $\pm$ 0.02 wt%, Lactic acid: 1.4 $\pm$ 0.03 wt%, Formic acid: 1.7 $\pm$ 0.05 wt% |
| Condensate biomethane yield | 508 $\pm$ 2.7 mL/g-VS | 471 $\pm$ 3.5 mL/g-VS | 493 $\pm$ 1.7 mL/g-VS |
| Condensate VS reduction after AD | ~35% | ~33% | ~31% |
| Condensate soluble COD reduction after AD | ~65% | ~60% | ~62% |

**5.3. Mass and energy distributions**

$Mass closure \left( \% \right)=\frac{Total output (Kg)}{Total mass input (Kg)}\times100$ ………Eqn. S1

$Energy closure \left( \% \right)=\frac{Total output (MJ)}{Total input (MJ)}\times100$………Eqn. S2

**5.4. Bio-methane potential (BMP) assay**

**S7. Buswell equation calculation for theoretical CH₄ yield from bamboo-derived condensate.**

| **Parameter / Calculation** | **Value** |
| --- | --- |
| Elemental composition (wt%, dry VS) | C = 44%, H = 7.3%, O = 48.7%, N ≈ 0% |
| Convert wt% to moles per 100 g VS | nC = 44 / 12 ≈ 3.667 mol;  nH = 7.3 / 1 = 7.3 mol;  nO = 48.7 / 16 ≈ 3.044 mol;  nN = 0 mol |
| Normalize to C = 1 | H/C = 7.3 / 3.667 ≈ 2  O/C = 3.044 / 3.667 ≈ 0.83  N/C = 0 |
| Molecular formula | **C_₁_H_₂_O_0.83_N_0_** |
| Buswell equation for CH₄ (moles per mole substrate) | nCH₄ = a/2 + b/8 - c/4 - 3d/8  nCH₄ = 1/2 + 2/8 - 0.83/4 ≈ 0.5425 mol CH₄/mol substrate |
| Molar mass of substrate (g/mol) | M = 12×1 + 1×2 + 16×0.83 = 27.28 g/mol |
| Volume of CH₄ per mole substrate (at STP) | V = 0.5425 × 22.4 L ≈ 12.15 L CH₄/mol substrate |
| CH₄ yield per gram VS | 12.15 L / 27.28 g ≈ 0.500 L/g VS ≈ 500 mL/g VS |

The theoretical CH₄ yield (~ 500 mL/g-VS) was estimated using the Buswell equation based on the elemental composition of bamboo-derived condensate (C_₁_H_₂_O_0.83_N_0_). The Buswell-based methane potential represents the stoichiometric limit, while COD-based scaling is used only to estimate the extent of experimental conversion.
